# Designer Enzymes: Food-System-Oriented Biocatalysts for Mitigating Mycotoxin Contamination and Reducing Postharvest Grain Loss

**DOI:** 10.3390/foods15193538

**Published:** 2026-10-04

**Authors:** Yiyang Zhang, Miao Long

**Affiliations:** Key Laboratory of Livestock Infectious Diseases, Ministry of Education, College of Animal Science and Veterinary Medicine, Shenyang Agricultural University, 120 Dongling Road, Shenyang 110866, China; 2025240884@stu.syau.edu.cn

**Keywords:** food and feed safety, sustainable food systems, mycotoxin detoxification, Designer Enzymes, artificial intelligence, multi-enzyme cascades, grain loss reduction, regulatory preparedness

## Abstract

Mycotoxin contamination remains a major challenge to food and feed safety, agricultural productivity, and postharvest resource utilization. Although numerous mycotoxin-transforming enzymes have been discovered and increasingly improved through protein engineering, only a limited number have progressed from laboratory characterization to practical food or feed applications. This translational gap arises because catalytic efficiency alone does not address matrix interference, processing stress, scalable production, formulation, degradation-product safety, and application-specific regulatory requirements. This review critically examines the development of enzyme-based detoxification across major mycotoxin classes, including aflatoxins, deoxynivalenol and related trichothecenes, zearalenone, ochratoxin A, and fumonisins, while explicitly distinguishing the deployment requirements of food and feed systems. Building on evidence from natural enzymes, engineered variants, matrix studies, animal evaluations, and commercially advanced systems, we use the term “Designer Enzymes” to describe a literature-derived, deployment-oriented engineering concept in which intended-use conditions are defined before optimization and used to guide molecular, manufacturing, formulation, safety, and regulatory decisions. A five-dimensional framework encompassing Mechanistic Understanding, Predictive Design Integration, Multi-Objective Optimization, Deployment Readiness, and Safety and Regulatory Preparedness is used as a qualitative diagnostic tool to identify translational bottlenecks rather than as a formally validated numerical scoring system. Representative systems targeting ZEN, DON, AFB1, OTA, and fumonisins are compared to illustrate their contrasting trajectories from mechanistic discovery to practical deployment. Structure-guided engineering, AI-assisted mutation prioritization, stability optimization, Design–Build–Test–Learn workflows, synthetic biology, multi-enzyme systems, scalable production, formulation, and existing pilot-scale or commercial translation are further evaluated according to the specific bottlenecks they are intended to address. Overall, this review argues that the future of enzymatic mycotoxin detoxification depends less on maximizing technological complexity than on integrating mechanistic evidence, application-specific performance, manufacturability, degradation-product safety, and regulatory readiness. The Designer Enzyme concept therefore provides a structured roadmap for directing promising detoxification enzymes toward credible, safe, and scalable food and feed applications.

## 1. Introduction

### 1.1. Mycotoxin Contamination as a Threat to Sustainable Food Systems

Mycotoxin contamination remains one of the most persistent threats to global food and feed safety. Produced primarily by fungal species belonging to the genera *Aspergillus*, *Fusarium*, and *Penicillium*, mycotoxins can contaminate cereals, oilseeds, nuts, animal feeds, and processed food products throughout production, storage, transportation, and commercialization [1,2]. Changes in temperature, drought frequency, precipitation patterns, and other environmental conditions can further alter fungal ecology and contamination patterns, increasing the complexity of mycotoxin management across agricultural supply chains [3,4,5].

Among the numerous mycotoxins identified to date, aflatoxin B1 (AFB1), deoxynivalenol (DON), zearalenone (ZEN), ochratoxin A (OTA), and fumonisins are of particular concern because of their widespread occurrence, toxicological significance, and economic impact [6]. These toxins differ substantially in chemical structure and biological activity and have been associated with genotoxic, immunotoxic, reproductive, nephrotoxic, hepatotoxic, and intestinal effects. Their structural diversity also means that detoxification cannot be achieved through a single universal reaction mechanism.

An additional challenge is that contamination frequently involves more than one toxin. Co-occurrence of multiple mycotoxins, together with modified or masked forms, can complicate analytical detection, risk assessment, and intervention strategies [7,8].

Enzymes that efficiently transform a single toxin under controlled conditions may therefore provide incomplete protection when commodities contain multiple chemically distinct contaminants. These realities make toxin specificity, degradation pathways, and application context central considerations in the development of enzymatic detoxification technologies.

Mycotoxin contamination also has implications beyond direct toxicological risk. Contaminated commodities may be rejected, downgraded from food to feed use, diverted to lower-value applications, or discarded, resulting in economic losses and inefficient use of agricultural resources. The historically cited estimate that approximately 25% of global crops are affected by mycotoxins has been widely discussed and subsequently re-evaluated, with actual contamination prevalence varying according to commodity, geographical region, analytical sensitivity, sampling strategy, and the threshold used to define contamination [1,2,3]. Regardless of the precise global prevalence, mycotoxin contamination can contribute to losses of already invested land, water, energy, labor, storage, and transportation resources.

Consequently, mycotoxin management should be viewed not only as a food- and feed-safety issue but also as a component of resource efficiency and postharvest loss reduction. Effective detoxification technologies must therefore reduce toxicological risk without introducing unacceptable losses in nutritional quality, product functionality, processing performance, or economic value.

### 1.2. Limitations of Conventional Detoxification and Enzymatic Approaches

A wide range of physical, chemical, and biological approaches has been developed to mitigate mycotoxin contamination. Physical interventions, including sorting, adsorption, irradiation, and thermal treatment, can reduce contamination under selected conditions but may be limited by incomplete toxin removal, loss of edible material, changes in nutritional or technological quality, or redistribution rather than destruction of the toxin [9].

Chemical detoxification can achieve substantial toxin conversion in some systems but may introduce concerns related to reaction products, residues, process severity, environmental burden, and consumer acceptance [10]. Biological detoxification has attracted increasing interest because it can operate under comparatively mild conditions and may provide greater reaction specificity [11]. Among biological approaches, enzymatic detoxification is particularly attractive because enzymes can directly modify toxicity-associated structural motifs while limiting non-specific changes to the surrounding food or feed matrix. A growing number of hydrolases, oxidoreductases, transferases, esterases, and other catalytic proteins have therefore been identified for the transformation of major mycotoxins. The major advantages, limitations, implementation challenges, and potential consequences of physical, chemical, natural enzymatic, and engineered enzyme-based mitigation strategies are comparatively summarized in Table 1.

Despite substantial progress in enzyme discovery and protein engineering, however, most reported mycotoxin-detoxifying enzymes have not progressed beyond laboratory-scale characterization. Common limitations include insufficient thermostability, narrow or poorly defined substrate scope, inadequate tolerance to complex matrices, low or inconsistent production yields, and incomplete characterization of degradation products [13,14]. These limitations become particularly important when enzymes are transferred from purified buffer systems to realistic food or feed environments.

The nature of this translational challenge differs substantially between food and feed applications. Food-processing enzymes must function within defined commodities and processing operations while preserving nutritional, sensory, and technological quality. Their performance can be affected by temperature, pH, water availability, proteins, lipids, polyphenols, and other matrix components. Feed-oriented enzymes may additionally encounter conditioning, pelleting, extrusion, drying, and storage before ingestion and, when gastrointestinal activity is intended, acidic conditions, digestive proteases, bile salts, intestinal enzymes, and other physiological stresses. These application-specific constraints are examined in detail in Section 2.

A critical disconnect therefore exists between biochemical proof of activity and practical detoxification. An enzyme may display high catalytic efficiency in purified buffer yet lose activity in a cereal matrix, fail during feed manufacture, exhibit insufficient expression for economical production, or generate transformation products whose toxicological properties remain uncertain. Moreover, improvement of one property does not necessarily solve the entire problem: enhanced catalytic activity may compromise stability, increased thermostability may not overcome poor toxin accessibility, and disappearance of the parent toxin does not establish detoxification when degradation products are insufficiently characterized.

The central translational challenge is therefore not simply a shortage of enzymes capable of transforming mycotoxins. Rather, it is the difficulty of simultaneously satisfying catalytic, processing, manufacturing, safety, and regulatory requirements under the conditions of intended use. These interconnected constraints explain why conventional sequential optimization frequently fails to generate application-ready detoxification systems and provide the rationale for a deployment-oriented engineering perspective.

### 1.3. From Natural Enzymes to Designer Enzymes

Recent advances in structural biology, computational modeling, artificial intelligence, protein engineering, and synthetic biology have expanded the range of strategies available for enzyme discovery and optimization [18,19]. Structure prediction and molecular modeling can support mechanistic interpretation [20], sequence-based and machine-learning approaches can prioritize candidate variants [21], and iterative Design–Build–Test–Learn (DBTL) workflows can connect computational hypotheses with experimental evidence. At the same time, advances in production-host engineering, fermentation, formulation, and modular biocatalysis have increasingly expanded enzyme development from molecular optimization toward system-level production and deployment [16].

For mycotoxin detoxification, however, the value of these technologies depends on whether they address the specific limitation preventing practical application. Structure-guided engineering may be appropriate when substrate recognition or catalytic mechanism is insufficiently understood, whereas stability-oriented computational design becomes relevant when processing robustness is limiting [22]. Production-host engineering may provide greater translational value when expression yield or manufacturing consistency is the dominant constraint, while formulation may be more appropriate when an otherwise suitable enzyme fails under processing or gastrointestinal conditions [23]. Thus, advanced engineering technologies should be regarded as complementary tools selected according to an identified bottleneck rather than as obligatory components of every enzyme-development program.

Within this context, we use the term “Designer Enzymes” to describe a deployment-oriented engineering concept in which the intended application is defined before optimization and is used to determine the required molecular, manufacturing, formulation, safety, and regulatory properties. The concept therefore does not refer simply to an enzyme carrying engineered mutations or to the use of a particular computational technology. Instead, it emphasizes the deliberate alignment of enzyme properties with the conditions under which detoxification is expected to occur.

This application-oriented perspective is particularly important because food and feed systems impose fundamentally different requirements. A food-oriented Designer Enzyme may need to retain activity in a specific commodity and processing operation while preserving sensory and technological quality. A feed-oriented system may instead require robustness during conditioning or pelleting, storage stability, gastrointestinal persistence where relevant, and evidence of target-animal efficacy [16,23,24]. Consequently, the same biochemical catalyst may exhibit different levels of deployment suitability depending on its intended application.

Drawing on recurring translational challenges reported across the literature [11], this review organizes existing evidence according to five interconnected dimensions: Mechanistic Understanding, Predictive Design Integration, Multi-Objective Optimization, Deployment Readiness, and Safety and Regulatory Preparedness. These dimensions form a literature-derived diagnostic framework for identifying evidence gaps and engineering priorities rather than a formally validated quantitative technology-readiness scale.

The resulting perspective shifts the central question from whether an enzyme can transform a mycotoxin under laboratory conditions to whether it can achieve meaningful toxicological risk reduction under the specific food or feed conditions for which it is intended. This distinction provides the conceptual basis for the Designer Enzyme framework developed in Section 3 and for the comparative analysis of representative enzyme systems in Section 4.

### 1.4. Scope and Objectives of This Review

This review critically examines the development of enzyme-based strategies for mitigating major mycotoxins and focuses on the factors that determine their progression from biochemical activity to practical food and feed application. The discussion encompasses representative systems targeting aflatoxins, deoxynivalenol and related trichothecenes, zearalenone, ochratoxin A, and fumonisins, with particular attention to catalytic mechanisms, protein engineering, matrix performance, scalable production, formulation, degradation-product safety, and regulatory translation. Earlier foundational studies are considered where necessary to establish the development of individual enzyme systems, whereas more recent literature is emphasized when assessing current engineering and deployment capabilities.

The scope of this review is restricted primarily to enzyme-based detoxification. Whole-cell microbial detoxification, adsorption technologies, plant resistance mechanisms, and other mitigation strategies are considered only when they provide mechanistic insight, enzyme candidates, comparative context, or technologies directly relevant to Designer Enzyme development. Rather than experimentally validating a new classification system, this review synthesizes heterogeneous published evidence to identify recurring translational bottlenecks and organize them within a deployment-oriented conceptual framework.

The review first examines toxin-specific structural determinants and the distinct processing and matrix constraints associated with food and feed applications (Section 2). It then defines the Designer Enzyme concept, introduces a prospective application-oriented design workflow, and presents a literature-derived five-dimensional framework for organizing evidence across Mechanistic Understanding, Predictive Design Integration, Multi-Objective Optimization, Deployment Readiness, and Safety and Regulatory Preparedness (Section 3). Representative enzyme systems targeting ZEN, DON and related trichothecenes, AFB1, OTA, and fumonisins are subsequently reviewed to illustrate contrasting development trajectories and identify the principal bottlenecks that remain unresolved (Section 4).

Section 5 and Section 6 then examine the engineering strategies that may be selectively applied to these bottlenecks. Structure prediction, mechanism-guided engineering, AI-assisted mutation prioritization, stability optimization, and DBTL workflows are considered at the molecular design level, whereas chassis engineering, scalable production, multi-enzyme systems, cell-free biocatalysis, formulation, and existing pilot-scale or commercial translation are discussed from a system-engineering perspective. Section 7 distinguishes food- and feed-specific deployment requirements and considers sustainability, food-loss reduction, regulatory readiness, and future development priorities.

Accordingly, this review provides three principal contributions. First, it integrates historical and current evidence across major classes of mycotoxin-detoxifying enzymes rather than focusing exclusively on newly emerging engineering technologies. Second, it provides a diagnostic framework for identifying whether the dominant limitation of an enzyme system lies in mechanistic understanding, predictive engineering, multi-objective performance, deployment validation, or safety and regulation. Third, it links these identified evidence gaps to application-specific engineering and validation strategies while explicitly distinguishing the requirements of food and feed systems.

**Figure 1:**
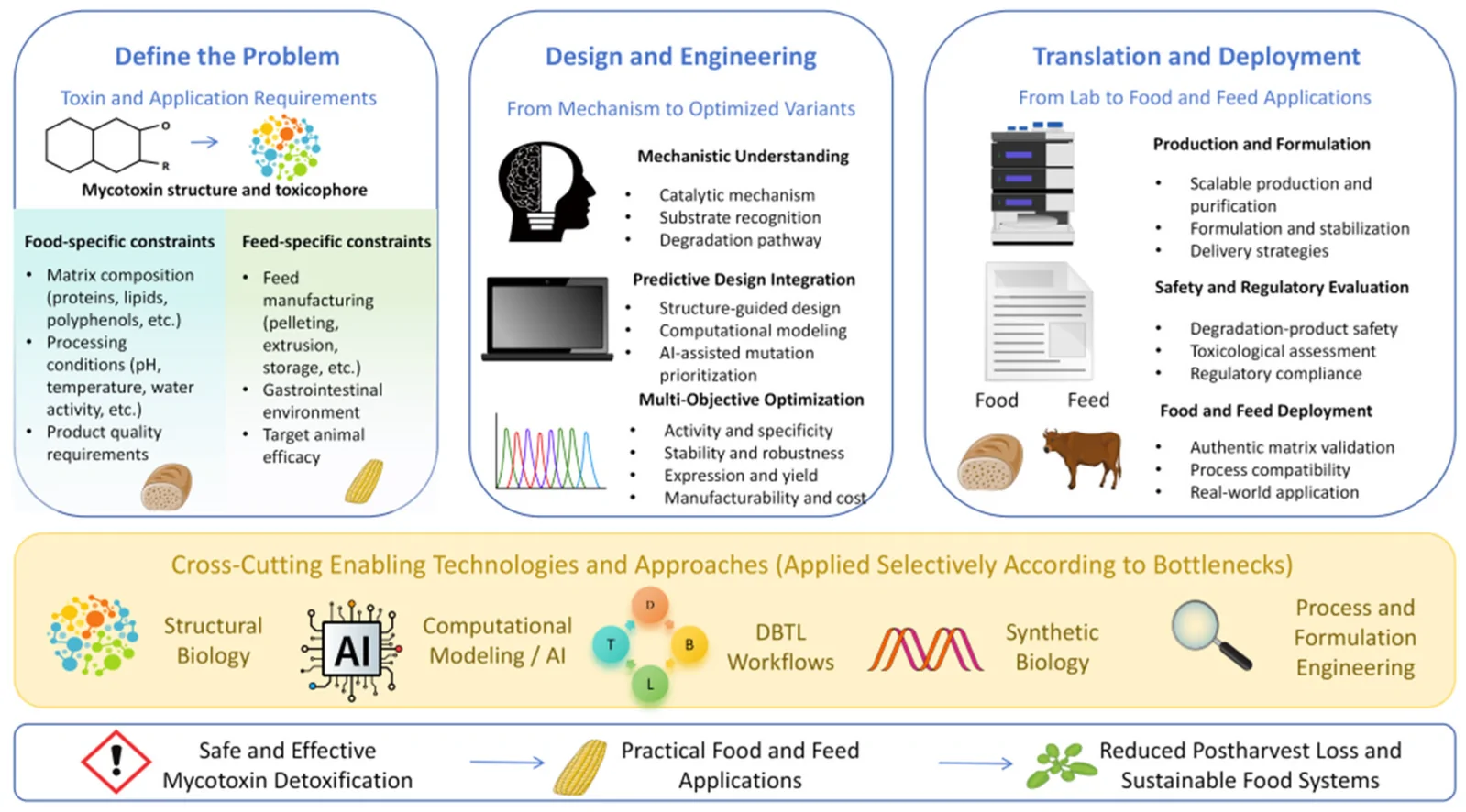
A Deployment-Oriented Framework for Designer Enzyme Development Against Mycotoxins.

The intended value of this review is therefore not merely to classify existing enzymes, but to provide a structured basis for determining what evidence or engineering step is required next to move a promising mycotoxin-detoxifying enzyme toward practical application. This deployment-oriented framework is illustrated graphically in Figure 1, which maps the three core development phases—Problem Definition, Design and Engineering, and Translation and Deployment—alongside the cross-cutting enabling technologies that support each stage.

## 2. Structural Characteristics of Major Mycotoxins and Application-Specific Constraints

### 2.1. Structural Toxicophores and Enzymatic Detoxification Targets

The structural diversity of mycotoxins fundamentally determines the enzymatic reactions required for detoxification because different toxins contain distinct toxicity-associated structural motifs. Effective detoxification therefore depends on selectively modifying these toxicophores while minimizing undesirable changes to the surrounding food or feed matrix [16,25]. For example, hydrolysis of the resorcyclic acid lactone ring is a well-established strategy for reducing the estrogenic activity of zearalenone [26,27], whereas detoxification of trichothecenes such as deoxynivalenol involves modification of toxicity-associated structural groups, including the 12,13-epoxide moiety [28]. Aflatoxin B1 has been targeted by several oxidoreductive enzyme systems, including laccases and other oxidoreductases, with transformations involving structural regions associated with its toxicity [25,29], while ochratoxin A can be detoxified through hydrolysis of the amide bond linking the isocoumarin moiety to phenylalanine [30,31]. Fumonisin detoxification, in contrast, can involve enzymatic hydrolysis of the tricarballylic acid side chains by fumonisin esterases such as FumD [32,33].

These mechanistic differences mean that no single catalytic strategy is universally applicable across major mycotoxin classes. Instead, toxin structure determines the required reaction chemistry, enzyme class, substrate-recognition mechanism, and the type of degradation products that must subsequently be evaluated. Structural understanding therefore provides the molecular basis for selecting appropriate enzyme scaffolds and for defining subsequent engineering objectives.

Within the Designer Enzyme concept discussed later in this review, toxicophore identification should be considered the starting point for mechanistic understanding rather than an isolated structural-analysis step. A clear definition of the toxicity-associated molecular target allows subsequent protein engineering to focus on catalytic selectivity, reaction efficiency, and degradation-product safety in a toxin-specific manner. Representative toxicophores, detoxification strategies, and enzyme systems for the major mycotoxins discussed in this review are summarized in Table 2.

### 2.2. Food-Specific Matrix and Processing Constraints

Food applications impose a set of constraints that differ substantially from those encountered in animal feed. In food systems, detoxification enzymes may be applied during grain treatment, milling, soaking, brewing, fermentation, baking, beverage processing, or other unit operations [38,39]. Under these conditions, enzyme performance is influenced not only by temperature and pH but also by the composition and physical properties of the food matrix. Proteins, lipids, carbohydrates, polyphenols, and limited water availability can alter toxin accessibility, promote non-specific enzyme adsorption, inhibit catalytic activity, or reduce molecular mobility [40]. Consequently, catalytic efficiency measured in purified buffer cannot be assumed to predict performance in authentic food matrices [41].

Processing operations introduce additional constraints. Thermal treatments may cause partial or complete enzyme inactivation, whereas changes in pH during fermentation or beverage production can shift enzymes away from their optimal catalytic range [11]. In low-moisture cereal matrices, restricted molecular mobility and mass transfer may limit contact between the enzyme and the target mycotoxin [42]. At the same time, food-processing applications require preservation of nutritional, sensory, and technological quality. Detoxification strategies should therefore avoid undesirable changes in color, flavor, texture, protein functionality, and other quality attributes of the final product [41,43].

These considerations also influence the timing of enzyme application. In some food processes, enzyme addition before a severe thermal step may be ineffective unless the catalyst is sufficiently thermostable or protected by formulation. In other cases, application after heating or during a moderate-temperature processing stage may provide a more practical intervention point. Therefore, food-oriented enzyme development should not focus on universal robustness alone, but should define performance requirements according to the specific commodity and processing stage in which detoxification is intended to occur.

From a translational perspective, food-specific deployment should therefore be evaluated according to matrix compatibility, processing stability, toxin accessibility, degradation-product safety, and preservation of product quality [12]. These requirements differ from those of feed applications and should be assessed independently rather than combined under a single generic “food/feed” criterion. The specific barriers and corresponding countermeasures across different food matrices and processing stages are summarized in Table 3.

### 2.3. Feed-Specific Processing and Gastrointestinal Constraints

Feed applications impose a distinct combination of manufacturing and gastrointestinal constraints. Before ingestion, detoxification enzymes may be exposed to thermal, mechanical, and moisture-related stresses during conditioning, pelleting, extrusion, drying, and storage [17,48]. Feed pelleting is particularly relevant because elevated temperature, moisture, and processing stress can reduce the retained activity of exogenous enzymes before ingestion. Consequently, thermostability and formulation stability represent important engineering requirements for feed-oriented detoxification enzymes.

Following ingestion, the enzyme encounters a highly dynamic gastrointestinal environment. Gastric acidity can reduce catalytic activity or destabilize enzyme structure, while proteolytic enzymes such as pepsin and trypsin may degrade the catalyst before detoxification is completed [49]. Bile-associated conditions, intestinal proteases, microbial activity, and feed-matrix components may further influence enzyme stability, toxin accessibility, and apparent detoxification performance [50]. Therefore, activity measured under simplified laboratory conditions cannot be assumed to predict detoxification performance during gastrointestinal transit or in the presence of authentic feed matrices [49].

Feed-oriented enzyme engineering should consequently consider both pre-ingestion processing stability and post-ingestion functionality. Potential strategies include thermostability engineering, acid-tolerant redesign, protease-resistant variants, encapsulation, immobilization, enteric protection, and controlled-release formulations [17,51]. The appropriate strategy depends on the intended site of detoxification. Enzymes designed to act before ingestion may prioritize processing stability and rapid toxin conversion in the feed matrix, whereas enzymes intended to function within the gastrointestinal tract require additional protection against acidic and proteolytic environments [52,53].

Feed deployment should therefore be evaluated using criteria that differ from those used for food applications. In addition to activity in authentic feed matrices, relevant evidence may include stability during manufacturing, storage performance, gastrointestinal persistence, release behavior, target-animal efficacy, and toxicological assessment of the resulting degradation products [54]. Treating these requirements separately from food-processing conditions provides a more realistic basis for evaluating deployment readiness. The major gastrointestinal barriers encountered by feed-oriented detoxification enzymes and the corresponding engineering strategies are summarized in Table 4.

### 2.4. Co-Contamination and Masked Mycotoxins Require System-Level Solutions

Practical contamination scenarios frequently involve more than one free mycotoxin. Food and feed commodities may contain multiple mycotoxins, modified derivatives, or masked forms that collectively increase detoxification complexity [16,57]. Because most detoxification enzymes exhibit relatively narrow substrate specificity, enzyme systems developed for a single toxin may provide incomplete risk reduction when co-contamination occurs [34].

Masked mycotoxins introduce an additional challenge because conjugation with plant-derived molecules can alter analytical detectability and biochemical accessibility without necessarily eliminating toxicological concern [58]. Enzymatic strategies for masked mycotoxins may therefore involve sequential reactions in which a deconjugating enzyme first converts the conjugated form into the parent toxin or a more accessible derivative, followed by a second detoxification enzyme targeting the exposed toxicophore [59]. Such strategies are particularly relevant to modified forms of DON and ZEN and illustrate why detoxification may need to be designed at the pathway level rather than around a single catalytic reaction [60].

Multi-enzyme systems provide one possible solution to these complex contamination patterns [61]. Existing approaches include enzyme cocktails, fusion proteins, modular enzyme assemblies, and cascade systems combining complementary catalytic functions [62,63]. These strategies can broaden catalytic coverage and enable simultaneous detoxification of multiple contaminants or sequential transformation of a single toxin through complementary reaction steps [61]. However, increasing system complexity also introduces additional engineering requirements, including compatibility among enzymes, overlapping reaction conditions, production cost, formulation stability, and characterization of multiple reaction products [17].

Accordingly, multi-enzyme detoxification should not be regarded as an obligatory feature of all Designer Enzymes but as an advanced strategy selected when contamination profiles justify system-level intervention. The design and implementation of such systems are discussed further in Section 6 in the context of synthetic biology, modular biocatalysis, and scalable production.

Collectively, these constraints demonstrate that mycotoxin-detoxifying enzymes cannot be evaluated solely according to catalytic efficiency measured under simplified laboratory conditions. Toxin structure determines the required reaction chemistry, whereas food and feed applications impose fundamentally different processing, matrix, physiological, and safety requirements. Co-contamination and masked mycotoxins further increase the need for toxin-specific and, where appropriate, system-level engineering strategies. These considerations provide the application context for the Designer Enzyme framework introduced in the following section, in which mechanistic understanding, predictive engineering, multi-objective optimization, deployment readiness, and safety preparedness are evaluated as interconnected components of enzyme translation.

## 3. Designer Enzymes: Definition and Literature-Derived Evaluation Framework

### 3.1. Why a Deployment-Oriented Framework Is Needed

Enzyme engineering for mycotoxin detoxification has advanced substantially over the past two decades, with numerous enzymes discovered and increasingly optimized through rational, structure-guided, and computational engineering approaches [16,19,43]. Nevertheless, only a limited number of these systems have progressed from laboratory characterization toward practical food or feed applications. This translational gap reflects the fact that catalytic activity alone is insufficient to determine whether an enzyme can function effectively under realistic deployment conditions [23,64].

Existing studies have often evaluated detoxification enzymes primarily according to isolated biochemical properties, such as catalytic efficiency, thermostability, pH tolerance, or substrate specificity [43]. These parameters remain essential, but practical deployment additionally requires evidence related to authentic food or feed matrices, processing conditions, manufacturing consistency, degradation-product safety, intended-use efficacy, and regulatory evaluation [23,64]. As a result, enzymes with promising laboratory performance may still fail to progress toward application because one or more deployment-related limitations remain unresolved.

Taken together, the literature indicates that enzyme translation involves multiple interconnected biochemical, manufacturing, application, safety, and regulatory requirements. An integrated framework may therefore help organize these heterogeneous forms of evidence across the enzyme-development pathway. Such an approach should not replace conventional biochemical characterization or established technology-readiness concepts. Rather, it should help identify where the principal translational bottleneck lies and which engineering step is most relevant for overcoming it.

In this review, we therefore use a deployment-oriented, literature-derived framework to synthesize current evidence across five interconnected dimensions: mechanistic understanding, predictive design integration, multi-objective optimization, deployment readiness, and safety and regulatory preparedness. The framework is intended as a heuristic diagnostic tool for comparing the strengths and limitations of different enzyme systems rather than as a formally validated quantitative classification method.

### 3.2. Operational Definition of Designer Enzymes

In this review, the term “Designer Enzymes” is used as an operational concept rather than as a rigid taxonomic category. It describes a shift in engineering philosophy from improving isolated enzyme properties toward designing biocatalytic systems according to predefined application requirements. A Designer Enzyme is therefore defined here as an enzyme or enzyme system whose development integrates catalytic-target selection, predictive engineering, experimental validation, scalable production, and application-oriented performance assessment within an iterative development process.

This definition does not imply that conventional enzyme engineering ignores stability, expression, formulation, or other practical considerations. Industrial enzyme development has long addressed such properties [65,66,67]. The distinction instead lies in when and how deployment-related requirements are incorporated into the engineering process. In many laboratory-scale enzyme-development studies, initial optimization focuses primarily on catalytic or biochemical properties, whereas application-related requirements may receive greater attention at later stages of development [68]. In a Designer Enzyme strategy, intended use conditions are defined at an earlier stage and are used to determine the engineering objectives from the outset.

Accordingly, the target properties of a Designer Enzyme depend on its intended application. A feed-oriented enzyme may require tolerance to feed processing and storage and, where gastrointestinal activity is intended, sufficient functionality under acidic and proteolytic conditions [23]. The same enzyme can therefore exhibit different levels of deployment suitability depending on whether it is intended for food or feed application [24].

**Figure 2:**
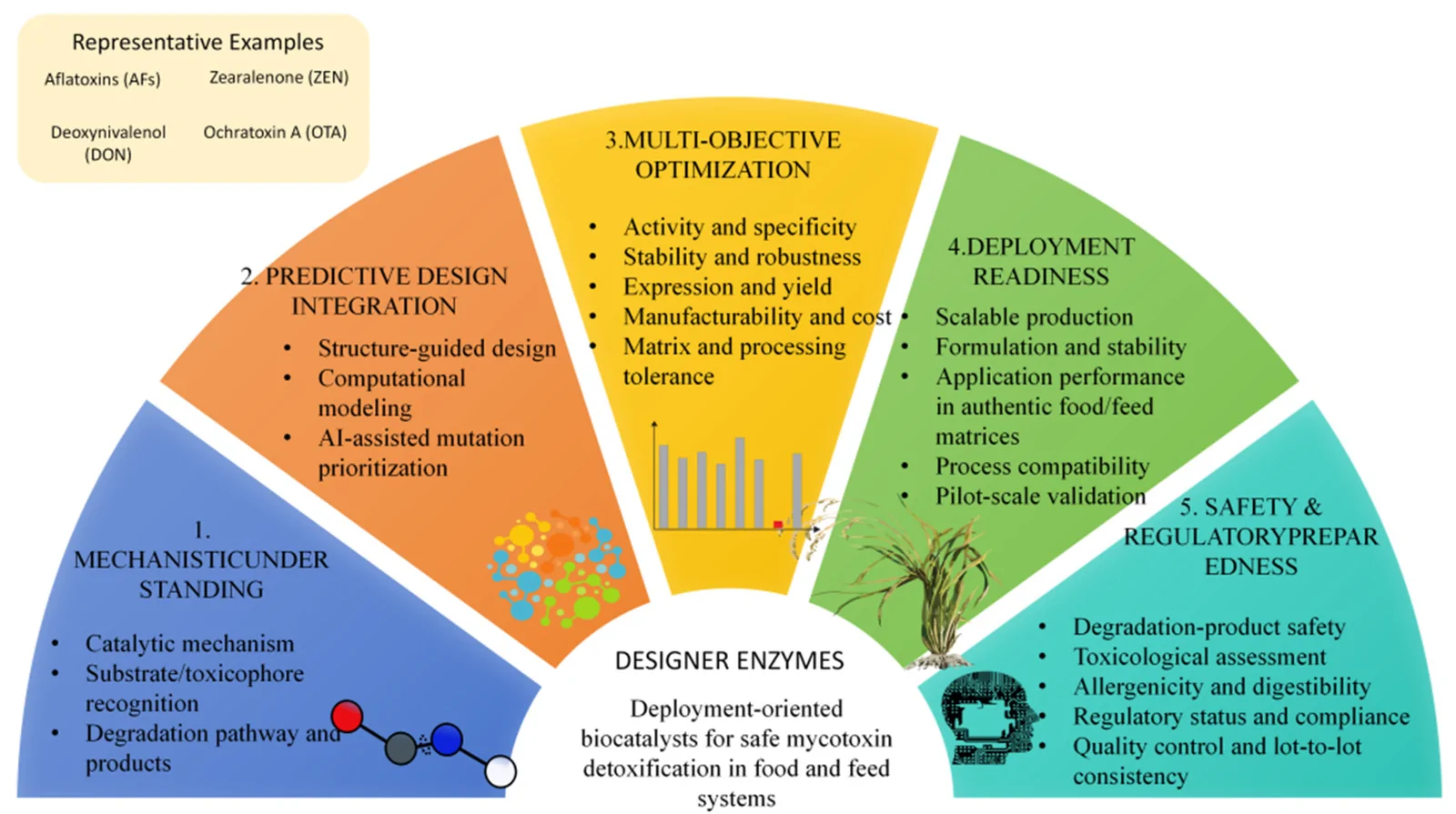
Five-Dimensional Literature-Derived Framework for Evaluating Designer Enzyme Development.

Designer Enzymes should consequently be viewed not simply as proteins carrying engineered mutations but as application-oriented biocatalytic systems in which molecular design, production strategy, formulation, safety evaluation, and deployment context are considered together. The practical value of this concept lies in making translational requirements explicit before optimization rather than evaluating them only after enzyme engineering has been completed. The five evidence dimensions that operationalize this Designer Enzyme concept—from mechanistic understanding through safety and regulatory preparedness—are illustrated in Figure 2, with representative mycotoxin targets shown across the top.

### 3.3. A Prospective Design Workflow for Designer Enzyme Development

The Designer Enzyme concept can be applied prospectively to guide enzyme development rather than only retrospectively to compare existing systems. The first step is to define the target mycotoxin, commodity, and intended point of intervention before selecting or modifying an enzyme scaffold. This initial definition determines the chemical transformation required, the relevant matrix constraints, and the performance criteria that should be prioritized during engineering.

A practical design workflow can be organized into six steps. First, the toxicity-associated structural motif of the target mycotoxin and the desired detoxification reaction are identified [16]. Second, an enzyme scaffold with an appropriate catalytic mechanism is selected from natural diversity, genomic or metagenomic resources, previously characterized enzyme families, or engineered variants [22]. Third, the intended application environment is defined, including food or feed matrix, temperature, pH, moisture, processing duration, and, where relevant, gastrointestinal exposure [11,38,49]. Fourth, the dominant translational bottleneck is identified and matched to an appropriate engineering strategy. Structure-guided mutagenesis may be prioritized when substrate recognition or catalytic interactions are limiting, whereas semi-rational or computational stability engineering may be selected when thermal robustness is inadequate, and production-host engineering may be prioritized when expression or manufacturing consistency is the major constraint [69]. Fifth, candidate enzymes are validated under progressively more realistic conditions, moving from purified systems to model matrices, authentic food or feed, and, where appropriate, pilot-scale processing [62,63]. Finally, degradation products, toxicological outcomes, manufacturing consistency, and regulatory requirements are evaluated before deployment.

The resulting experimental evidence can be incorporated into iterative Design–Build–Test–Learn cycles, allowing engineering objectives to be updated as new bottlenecks are identified [20]. Importantly, the Designer Enzyme concept does not require the simultaneous use of artificial intelligence, automated DBTL, synthetic biology, and every available engineering technology. Instead, the choice of tools should be driven by the specific limitation preventing translation.

Thus, the “design” component of the Designer Enzyme concept lies in defining deployment requirements before optimization and using these requirements to determine which molecular, production, formulation, and safety properties need to be engineered.

### 3.4. Five-Dimensional Literature-Derived Evaluation Framework

To organize the diverse evidence required for practical translation, current studies can be interpreted across five complementary dimensions: Mechanistic Understanding, Predictive Design Integration, Multi-Objective Optimization, Deployment Readiness, and Safety and Regulatory Preparedness. These dimensions reflect recurring bottlenecks identified across the literature rather than a universally validated technology-readiness scale.

Mechanistic Understanding addresses whether the catalytic target, key residues, reaction pathway, substrate-recognition mechanism, and major transformation products have been adequately characterized. Strong mechanistic evidence provides the molecular basis for rational engineering and reduces uncertainty regarding the relationship between toxin transformation and detoxification [25].

Predictive Design Integration considers the extent to which structural information, molecular modeling, simulation, sequence-function relationships, or artificial-intelligence-assisted approaches are used to guide engineering decisions. The purpose of this dimension is not to reward computational complexity itself, but to assess whether predictive information meaningfully reduces empirical search space and improves engineering efficiency.

Multi-Objective Optimization evaluates whether enzyme development addresses multiple application-relevant properties simultaneously. Depending on the intended application, these may include catalytic efficiency, thermostability, pH tolerance, expression yield, substrate specificity, matrix tolerance, protease resistance, formulation compatibility, or manufacturability. Because improvement in one property can compromise another, balanced optimization is more informative than isolated enhancement of a single biochemical metric [70,71].

Deployment Readiness concerns evidence obtained under conditions that approximate intended use. For food applications, this may include authentic food matrices, processing temperature, pH, residence time, product-quality effects, and compatibility with unit operations. For feed applications, deployment evidence may additionally include conditioning and pelleting stability, storage performance, gastrointestinal persistence, controlled release, and target-animal efficacy. Pilot-scale and commercial data provide stronger evidence of deployment readiness than buffer-based laboratory assays alone.

Safety and Regulatory Preparedness evaluates whether degradation products, production organisms, enzyme preparations, toxicological outcomes, and intended-use conditions have been sufficiently characterized to support practical application. Importantly, disappearance of the parent mycotoxin does not by itself demonstrate detoxification. Product identification and biological safety therefore represent independent requirements that cannot be substituted by catalytic performance.

These five dimensions are interconnected rather than strictly sequential. Strong mechanistic understanding may facilitate predictive engineering, while multi-objective optimization may improve deployment performance. However, high maturity in one dimension cannot fully compensate for major deficiencies in another. For example, excellent catalytic activity and structural understanding cannot substitute for insufficient degradation-product safety or lack of validation in the intended food or feed system. The key distinctions between conventional enzyme optimization and this deployment-oriented Designer Enzyme approach are summarized in Table 5.

### 3.5. Application-Specific Interpretation of the Framework

The five dimensions should be interpreted according to the specific mycotoxin, enzyme class, and intended application rather than through universal numerical thresholds. For example, thermal resistance may be a dominant requirement for an enzyme intended to survive feed pelleting, whereas the same thermal threshold may have limited relevance to an enzyme applied during a low-temperature food-processing step. Similarly, gastrointestinal stability is central to enzymes intended for in vivo feed detoxification but is not an appropriate maturity criterion for an enzyme used during food fermentation or grain washing.

Toxin chemistry also affects interpretation of the framework. Hydrolytic systems such as ZEN lactonases may allow relatively direct linkage between defined bond cleavage and reduced toxicity, whereas oxidative detoxification of AFB1 can generate multiple intermediates and may therefore require more extensive product characterization before comparable safety confidence can be achieved. Likewise, enzymes targeting masked or co-occurring mycotoxins may require system-level validation that is unnecessary for a highly specific single-toxin application.

Accordingly, maturity should be interpreted as an evidence profile rather than as a single absolute score. A system may be mechanistically advanced but deployment-poor, or commercially established despite comparatively limited predictive redesign. Such differences are scientifically informative because they identify which part of the translation pathway remains incomplete.

In the representative case studies discussed in Section 4, the framework is therefore used primarily to compare evidence strengths, weaknesses, and translational bottlenecks. Any numerical values retained for visualization should be regarded as illustrative summaries of published evidence rather than validated quantitative measures of technological readiness. The five evidence dimensions and their progression from early to advanced stages are operationally defined in Table 6 to support structured literature-based assessment.

### 3.6. Toxicological Assessment of Enzymatic Degradation Products

Reduction in the concentration of a parent mycotoxin should not be interpreted automatically as successful detoxification. Enzymatic reactions may generate intermediates or final products with residual biological activity, and in some cases the toxicological properties of these compounds remain insufficiently characterized. Consequently, analytical confirmation of toxin disappearance should be complemented by identification of transformation products and assessment of their biological safety.

The strength of toxicological evidence differs substantially among enzyme–mycotoxin systems. ZEN lactonases provide one of the clearest examples in which hydrolysis of the resorcyclic acid lactone ring is associated with formation of products exhibiting markedly reduced estrogenic activity [26,27,72]. OTA hydrolysis produces ochratoxin α and phenylalanine, with the former generally considered substantially less toxic than the parent compound [73,74,75]. Fumonisin esterases such as FumD generate hydrolyzed fumonisins, including hydrolyzed fumonisin B1 (HFB1), which have been evaluated in toxicological, toxicokinetic, and regulatory assessments [35]. In contrast, oxidative transformation of AFB1 can generate multiple structurally diverse products, and toxicological characterization remains incomplete for many enzyme–reaction systems [76,77].

A robust safety-evaluation strategy should therefore combine chemical and biological evidence. Relevant approaches include LC–MS/MS or high-resolution mass spectrometry for product identification, in vitro cytotoxicity or mechanism-relevant bioassays, genotoxicity assessment where appropriate, and animal studies when required for the intended use and regulatory pathway. The depth of testing should be proportional to the expected exposure, application route, enzyme source, and uncertainty associated with the degradation products.

Within the Designer Enzyme framework, degradation-product safety is not a secondary endpoint but a core component of practical translation. An enzyme should therefore be considered deployment-ready only when catalytic efficacy and toxicological risk reduction are supported by complementary evidence.

### 3.7. Limitations of the Proposed Framework

The proposed framework has several limitations that should be considered when interpreting the comparative analyses presented in this review. First, the framework is derived from heterogeneous published literature in which experimental protocols, enzyme preparations, toxin concentrations, matrices, and reporting standards vary considerably. Direct comparison across enzyme systems is therefore inevitably constrained by differences in evidence quality and experimental context.

Second, the five dimensions are not associated with universally applicable quantitative thresholds. Requirements vary according to mycotoxin chemistry, enzyme class, food or feed application, processing conditions, and regulatory jurisdiction. For this reason, the framework should be interpreted as a diagnostic structure for organizing evidence rather than as a standardized scoring instrument.

Third, the framework emphasizes translational maturity and may therefore underestimate the scientific importance of early-stage mechanistic discoveries. A newly identified enzyme with exceptional catalytic novelty may have limited deployment evidence while still providing an important foundation for future engineering. Low deployment maturity should therefore not be interpreted as low scientific value.

Fourth, the framework has not undergone formal validation through expert consensus, inter-laboratory benchmarking, or correlation with established technology-readiness systems. Any comparative maturity designation used in representative case studies should therefore be regarded as qualitative and illustrative rather than definitive. Future refinement may benefit from standardized datasets, expert consensus exercises, and harmonized reporting criteria across mycotoxin-detoxification studies.

Finally, the framework is intended to complement rather than replace conventional Technology Readiness Level concepts. General TRL systems provide a useful indication of technological progression but do not explicitly capture several issues that are particularly important for mycotoxin-detoxifying enzymes, including food-matrix compatibility, feed-processing and gastrointestinal functionality, degradation-product toxicology, and application-specific regulatory requirements.

Despite these limitations, the framework provides a structured way to identify why apparently promising enzymes remain difficult to translate and which type of evidence or engineering intervention is required next. Representative examples are discussed in the following section.

## 4. Representative Mycotoxin-Detoxifying Enzymes: Existing Evidence and Translational Maturity

The development of mycotoxin-detoxifying enzymes has progressed unevenly across different toxin classes, with some systems remaining predominantly at the biochemical or mechanistic stage and others advancing to matrix validation, animal studies, regulatory assessment, or commercial deployment. Comparing these trajectories provides a useful overview of how the field has evolved from enzyme discovery toward practical detoxification technologies.

This section therefore reviews representative enzyme systems targeting major mycotoxins discussed in Section 2, including ZEN, DON and related trichothecenes, AFB1, OTA, and fumonisins. Rather than treating these examples as formal validation of the five-dimensional framework, the framework is used as a qualitative lens to organize published evidence and identify the principal translational bottlenecks associated with each system. Particular attention is given to the distinction between mechanistic knowledge, engineering progress, matrix validation, scale-up, degradation-product safety, and regulatory evidence.

The selected examples are not intended to represent an exhaustive catalogue of all known mycotoxin-detoxifying enzymes. Instead, they illustrate contrasting development trajectories, ranging from structurally well-characterized but application-limited enzymes to systems that have already progressed into regulated feed applications.

### 4.1. ZHD101 and Related ZEN Lactonases

ZHD101 and related zearalenone lactonases represent one of the most extensively characterized classes of mycotoxin-detoxifying enzymes. Early work established that ZHD101 from *Clonostachys rosea* catalyzes hydrolysis of the resorcyclic acid lactone ring of ZEN, generating products with substantially reduced estrogenic activity. Subsequent structural and biochemical studies identified the catalytic triad, substrate-binding pocket, key interacting residues, and structural determinants governing substrate recognition. These studies established a strong mechanistic foundation for subsequent structure-guided enzyme engineering [26,78].

More recent research has shifted from enzyme discovery toward improving properties relevant to practical application. Structure-guided mutagenesis, stability engineering, sequence optimization, and screening of related hydrolases have been used to improve catalytic efficiency, thermostability, acid tolerance, and activity in cereal-based matrices [22,69,79]. This progression illustrates a broader transition within the ZEN-detoxification field from identifying naturally active enzymes toward engineering catalysts that can retain performance under processing-relevant conditions.

The relatively well-defined hydrolytic mechanism of ZEN lactonases provides an important advantage for rational engineering. Because ZEN detoxification by lactonases involves a defined ring-opening reaction, mechanistic changes can be linked relatively directly to identifiable products and biological outcomes, in contrast to some oxidative detoxification systems that generate multiple products. In addition, evidence indicating markedly reduced estrogenic activity of hydrolysis products provides stronger support for true detoxification rather than disappearance of the parent toxin alone [27].

Despite this mechanistic and engineering progress, the maturity of ZEN lactonase systems remains strongly application- and enzyme-dependent. Recent studies have extended validation beyond purified systems into cereal-derived food or feed matrices, and at least some ZEN hydrolase systems have progressed to formal regulatory assessment for feed use. Nevertheless, large-scale processing validation, formulation stability, long-term storage, manufacturing consistency, and cross-matrix reproducibility remain less extensively documented for many ZHD-family enzymes than their biochemical and structural properties. Accordingly, regulatory progress observed for specific systems such as ZenA should not be generalized to all ZEN lactonases.

Current evidence consequently positions ZHD101-related lactonases as highly informative models for mechanism-guided Designer Enzyme development. For the best-characterized ZHD-family systems, the major translational challenge has shifted from identification of the core catalytic reaction toward demonstrating robust, reproducible performance under clearly defined food or feed deployment conditions.

### 4.2. Fhb7 and Trichothecene-Detoxifying Enzymes

*Fhb7* provides a mechanistically distinctive example of trichothecene detoxification. The gene was originally identified in the context of *Fusarium* head blight resistance in wheat, where it was shown to encode a glutathione S-transferase associated with broad resistance to *Fusarium*-derived trichothecenes [35]. Subsequent biochemical work demonstrated that Fhb7-GST catalyzes glutathione-dependent transformation of type A, B, and D trichothecenes into glutathione adducts with substantially reduced toxicity [80]. This discovery established an important link between natural disease resistance and enzymatic detoxification of DON and related trichothecenes.

Compared with ZEN lactonases, however, the development trajectory of Fhb7 has remained more closely associated with plant resistance biology than with industrial enzyme application. Existing studies provide strong evidence for biological function, substrate transformation, and reduced toxicity of major glutathione conjugates, whereas published evidence on high-yield stand-alone enzyme production, formulation, processing stability, and validation in naturally contaminated food or feed matrices remains comparatively limited.

This distinction is important when interpreting technological maturity. Fhb7 should not be considered a weak enzyme system simply because industrial evidence is limited. Rather, Fhb7 represents a system in which mechanistic and toxicological evidence has advanced more rapidly than stand-alone production and deployment engineering. Its current scientific value therefore lies in providing a well-defined biochemical strategy that may serve as a starting point for subsequent optimization.

Future development toward a stand-alone application-oriented biocatalyst would require more systematic characterization of expression yield, thermostability, pH tolerance, processing stability, matrix performance, formulation, and manufacturing scalability, while existing transformation-product safety evidence should be extended to application-relevant exposure scenarios. Structure-guided engineering or predictive sequence design may also be used to improve properties that were not primary objectives of the original plant-resistance studies. Importantly, such work would represent a transition from using *Fhb7* as a resistance-associated gene toward developing it as a stand-alone food- or feed-oriented biocatalyst.

Fhb7 therefore illustrates a recurring pattern in the mycotoxin-detoxification field: strong mechanistic evidence can provide an important foundation for Designer Enzyme development even when manufacturing and deployment data remain at an early stage.

### 4.3. CotA and Other AFB1-Transforming Oxidoreductases

Enzymatic transformation of AFB1 has been investigated using several classes of oxidoreductases, including laccases, peroxidases, and other oxidative enzymes. Among these, bacterial CotA-type laccases have attracted attention because of their relative structural robustness, multicopper catalytic architecture, and capacity to oxidize structurally diverse substrates. CotA-based systems therefore provide a useful model for examining both the opportunities and limitations associated with oxidative mycotoxin detoxification [81].

Previous studies have demonstrated that AFB1-degrading bacterial laccases can be improved through screening, site-directed or saturation mutagenesis, structure-guided engineering, and expression-system optimization [82]. Recent work has also demonstrated CotA systems capable of transforming both AFB1 and ZEN, including mediator-assisted and immobilized configurations, providing proof-of-concept for broadened substrate coverage under co-contamination scenarios [83,84]. These advances indicate that oxidative enzymes can be adapted beyond their native substrate ranges through targeted engineering.

However, oxidative detoxification presents a more complex mechanistic challenge than hydrolytic cleavage of a single well-defined bond. Laccase-catalyzed reactions can involve electron-transfer processes, radical intermediates, mediator effects, and multiple transformation products [85,86]. Consequently, disappearance of AFB1 does not by itself provide sufficient evidence that the resulting products are biologically safe. Identification of reaction products and evaluation of residual mutagenic, genotoxic, or cytotoxic activity remain particularly important for this enzyme class.

In authentic food or feed matrices, CotA performance may additionally be influenced by matrix composition, redox-active components, substrate accessibility, and water availability, making real-matrix validation essential. The broad substrate scope characteristic of laccases may also create a selectivity challenge because competing oxidizable matrix components could divert catalytic activity or alter product quality. Therefore, food-oriented development of CotA-derived systems should evaluate not only toxin removal but also substrate selectivity, matrix compatibility, degradation-product safety, and potential effects on food quality.

CotA therefore represents a valuable but mechanistically more complex platform for Designer Enzyme development. For well-characterized CotA systems, future translational progress is likely to depend as much on reaction selectivity, product safety, matrix compatibility, formulation, and production performance as on further increases in nominal AFB1 degradation activity.

### 4.4. OTA-Degrading Hydrolases

Ochratoxin A provides an important complementary example of hydrolytic mycotoxin detoxification. The molecule contains an amide bond linking an isocoumarin moiety to phenylalanine, and cleavage of this bond yields ochratoxin α (OTα) and phenylalanine [30]. Because OTα exhibits substantially lower toxicity than the parent toxin, enzymatic hydrolysis has long been regarded as one of the most promising routes for OTA detoxification [87].

A range of enzymes, including carboxypeptidases, amidases, and specialized amidohydrolases, have been investigated for OTA degradation. Earlier studies largely focused on screening microorganisms or crude enzymatic preparations for OTA-transforming activity, whereas more recent work has increasingly resolved individual catalytic proteins and their molecular mechanisms. Structural characterization of highly active OTA-detoxifying amidohydrolases has further enabled rational engineering of substrate recognition and catalytic performance.

These developments illustrate how the OTA field is progressing from activity-based discovery toward mechanism-guided optimization. Nevertheless, high catalytic efficiency in purified systems does not automatically establish suitability for food applications. OTA frequently occurs in cereals, coffee, wine-related products, dried fruits, and other chemically complex matrices in which pH, polyphenols, proteins, and processing conditions may alter enzyme performance. Consequently, matrix-specific validation remains essential.

Product safety should also be considered explicitly. Although hydrolysis to ochratoxin α generally provides a clearer detoxification endpoint than poorly defined oxidative transformation, residual substrate, secondary reactions, enzyme-source safety, and exposure to the resulting products still require appropriate evaluation according to the intended application.

OTA-degrading hydrolases therefore represent an increasingly mature mechanistic platform with strong potential for Designer Enzyme development. Their next translational priorities include application-specific stability engineering, scalable production, validation in authentic food matrices, and integration of degradation-product safety with process performance.

### 4.5. FumD and Fumonisin Esterases

Fumonisin esterases represent one of the clearest examples of enzymatic mycotoxin detoxification progressing beyond laboratory proof-of-concept toward practical feed application [23,24,64]. FumD and related carboxylesterases hydrolyze the tricarballylic acid side chains of fumonisins, generating hydrolyzed derivatives with substantially reduced toxicological activity [88]. This relatively well-defined reaction provides both a biochemical basis for detoxification and a measurable endpoint for efficacy evaluation.

In contrast to many mycotoxin-detoxifying enzymes that remain primarily at laboratory scale, fumonisin esterase systems have advanced through feeding studies, safety assessments, manufacturing characterization, regulatory evaluation, and ultimately authorized feed-additive use in animal nutrition [23]. This development history is particularly valuable because it demonstrates that successful translation requires substantially more than identification of an active enzyme. Evidence relating to production-organism safety, manufacturing consistency, efficacy in contaminated feed, target-animal tolerance, transformation-product safety, and intended conditions of use must also be assembled before commercialization.

The FumD case therefore provides an important counterpoint to more mechanistically sophisticated but less deployed enzyme systems. Detailed computational redesign or high-resolution mechanistic information is not necessarily a prerequisite for practical impact when efficacy, safety, production, and regulatory requirements have been sufficiently addressed. Conversely, advanced structural or computational knowledge cannot compensate for an absence of deployment evidence.

Because FumD has already reached comparatively advanced feed applications, its remaining development priorities differ from those of early-stage enzymes. Further improvement may focus on formulation efficiency, stability under diverse feed-manufacturing conditions, dose optimization, broader validation across feed matrices, and adaptation to jurisdiction-specific regulatory requirements. These goals represent refinement of an existing deployment platform rather than the initial establishment of industrial feasibility.

FumD consequently provides one of the strongest existing demonstrations of the central principle underlying the Designer Enzyme concept: practical maturity is determined by the integration of catalytic efficacy with manufacturability, matrix performance, toxicological safety, and regulatory readiness rather than by molecular engineering sophistication alone.

### 4.6. Comparative Translational Lessons from Representative Enzyme Systems

Comparison of the representative systems reviewed above reveals that mycotoxin-detoxifying enzymes do not progress through translation along a single uniform pathway. Instead, different systems exhibit distinct evidence profiles that reflect their discovery history, catalytic mechanism, intended application, and degree of industrial development.

ZHD101-related lactonases represent a mechanism-rich platform in which a defined toxicophore, well-characterized hydrolytic reaction, and substantial protein-engineering effort provide a strong basis for rational optimization [26]. Their principal remaining challenges lie increasingly in process-specific validation, scalable formulation, and broader application-specific regulatory translation. Fhb7 represents a different trajectory: mechanistic and biological evidence is comparatively strong, but its development as a stand-alone industrial detoxification enzyme remains limited because most work has focused on crop resistance rather than production and processing [35].

CotA-derived laccases illustrate the additional uncertainty associated with oxidative detoxification. Their broad catalytic capacity provides opportunities for activity against AFB1 and potentially multiple substrates, but this same catalytic breadth increases the importance of product identification, reaction selectivity, food-matrix effects, and toxicological validation. OTA hydrolases, in contrast, benefit from a relatively clear hydrolytic detoxification route, and recent structural studies provide an increasingly strong basis for mechanism-guided engineering, although authentic food-matrix and deployment evidence remain comparatively less developed.

FumD represents the most deployment-oriented example among the systems considered here because fumonisin esterase technology has progressed into regulated feed applications. Its trajectory demonstrates that translational maturity can be achieved through effective integration of efficacy, production, safety, and regulatory evidence even when computational redesign is less extensive than in some laboratory-focused enzyme systems.

Taken together, these examples demonstrate that no single indicator can adequately define the maturity of a mycotoxin-detoxifying enzyme. Mechanistic resolution, computational sophistication, catalytic efficiency, real-matrix validation, manufacturing feasibility, and regulatory preparedness contribute different forms of evidence and should be interpreted in relation to the intended application. The primary value of the five-dimensional framework is therefore to identify the dominant bottleneck for each enzyme–mycotoxin–application combination rather than to generate an absolute ranking of enzymes.

These comparative lessons also clarify the role of design in future development. A mechanistically mature but deployment-limited enzyme should not necessarily undergo further catalytic optimization; resources may instead be directed toward formulation, process integration, or safety assessment. Conversely, an industrially promising enzyme with poorly understood selectivity may benefit from structural and computational redesign. Designer Enzyme development therefore requires matching the engineering strategy to the specific evidence gap revealed by the existing literature. The comparative evidence profiles and dominant translational bottlenecks of the five representative enzyme systems are summarized in Table 7.

Having established the contrasting translational trajectories of representative detoxification enzymes, the following section examines the computational and AI-assisted engineering strategies that can be selectively applied to address these bottlenecks.

## 5. Computational Biology and AI-Assisted Strategies for Designer Enzyme Development

The representative enzyme systems discussed in Section 4 demonstrate that different mycotoxin-detoxifying enzymes are limited by different engineering bottlenecks. Some systems lack sufficiently resolved catalytic mechanisms, whereas others require improved stability, substrate selectivity, expression performance, or adaptation to realistic food and feed environments. Computational biology and artificial intelligence provide complementary tools for addressing these limitations by reducing experimental search space and prioritizing engineering hypotheses before extensive laboratory screening [18,19,90].

Importantly, computational methods should not be regarded as obligatory components of every Designer Enzyme program. Their value depends on whether a specific translational bottleneck can be addressed through predictive analysis. Structure prediction may be most useful when experimentally determined structures are unavailable, molecular modeling can support interpretation of enzyme–substrate interactions, and machine-learning or sequence-based approaches may assist mutation prioritization when large sequence spaces must be explored [19]. In all cases, computational predictions require experimental validation under conditions relevant to the intended application.

Within the five-dimensional framework described in Section 3, computational approaches primarily strengthen Mechanistic Understanding, Predictive Design Integration, and Multi-Objective Optimization. Their practical contribution is therefore not the use of advanced algorithms per se, but the extent to which they improve engineering decisions and reduce inefficient experimental iteration. Representative computational approaches and their roles in Designer Enzyme development are summarized in Table 8.

### 5.1. Structure Prediction and Mechanism-Guided Design

Structural information provides an important foundation for mechanism-guided enzyme engineering because it can help identify catalytic residues, substrate-recognition regions, access channels, and candidate sites for modification [30]. However, experimentally determined structures remain unavailable for many mycotoxin-detoxifying enzymes, limiting the direct application of structure-based engineering approaches. Structure-prediction methods therefore provide useful models for generating experimentally testable hypotheses when high-resolution structural data are lacking [92].

For Designer Enzyme development, the value of predicted structures lies primarily in supporting engineering decisions rather than replacing experimental structural biology. Predicted models can be used to propose catalytic residues, analyze possible toxin-binding regions, compare homologous enzymes, and identify candidate mutations for subsequent experimental testing [93]. Such information is particularly relevant when the primary limitation is incomplete mechanistic understanding or uncertain substrate recognition.

Nevertheless, predicted structures should be interpreted cautiously. Enzymes requiring metal centers, cofactors, flexible loops, post-translational modifications, or complex conformational transitions may not be fully represented by static structural models [70]. In addition, a plausible docking configuration does not establish a catalytic mechanism unless supported by biochemical, mutational, kinetic, or product-characterization evidence.

Structure prediction should therefore be treated as a hypothesis-generating component of mechanism-guided design. When combined with experimental validation, it can strengthen Mechanistic Understanding and Predictive Design Integration while reducing the number of candidate positions requiring empirical investigation.

### 5.2. AI-Assisted Mutation and Sequence Prioritization

Artificial-intelligence-assisted protein engineering offers additional opportunities for prioritizing potentially beneficial mutations or sequence variants before experimental screening [19]. Protein language models and other sequence-based predictive approaches can identify statistical relationships between protein sequence and functional constraints, whereas structure-conditioned sequence-design methods can propose sequences compatible with a predefined protein fold [91].

Within a Designer Enzyme workflow, the most immediate value of these approaches lies in reducing the size of the experimental search space. Rather than generating large mutation libraries without prior prioritization, computational models can be used to rank candidate substitutions according to predicted effects on stability, structural compatibility, or other relevant properties. Selected variants can then be tested experimentally, and the resulting data can be used to refine subsequent design decisions.

The appropriate use of AI should remain problem-specific. For an enzyme with poor thermal stability but well-defined catalytic chemistry, computational prioritization may preferentially explore substitutions predicted to improve stability while preserving catalytic-site geometry. This targeted strategy reduces the risk of compromising activity while systematically addressing the primary deployment bottleneck. For an enzyme such as a broad-specificity oxidoreductase, predictive approaches may instead be used to explore substrate-recognition regions or mutations associated with improved selectivity. In contrast, when the major limitation is large-scale production or formulation rather than protein sequence, further computational redesign may provide limited translational benefit [94,95].

Advanced AI-based protein design remains less extensively documented for mycotoxin-detoxifying enzymes than for protein engineering more broadly. Consequently, these methods should presently be regarded as promising engineering tools rather than established solutions for mycotoxin detoxification. Computationally prioritized variants require experimental validation. For deployment-oriented development, this validation should subsequently extend to relevant food or feed matrices and intended-use conditions.

### 5.3. Multi-Objective Optimization and Stability Engineering

Practical mycotoxin detoxification rarely depends on a single enzyme property. Improvements in catalytic activity may be insufficient if the enzyme is unstable during processing, poorly expressed, inhibited by food components, or unable to function within the intended pH range [11,79]. Designer Enzyme development therefore requires consideration of multiple objectives, including catalytic efficiency, stability, substrate specificity, expression performance, matrix compatibility, and manufacturability.

Computational approaches can support this process by identifying mutations that are predicted to improve one property while minimizing adverse effects on others. Stability-oriented design is particularly relevant because insufficient thermostability or pH robustness remains a recurring limitation among mycotoxin-detoxifying enzymes [69]. Structure-guided stability tools can prioritize substitutions predicted to stabilize the protein fold, after which experimental testing determines whether catalytic competence is retained.

PROSS and FRESCO provide representative examples of computationally assisted stability engineering. PROSS uses structural information together with sequence-based constraints to prioritize substitutions predicted to improve protein stability while limiting disruption of the existing fold [96]. FRESCO similarly combines computational prediction of stabilizing mutations with experimental screening and subsequent combination of beneficial substitutions [97]. In both cases, computation is used to reduce the number of variants requiring laboratory testing rather than to replace experimental evaluation [98].

These strategies are relevant to mycotoxin-detoxifying enzymes when stability represents a clearly identified deployment bottleneck. For example, ZEN-degrading hydrolases intended for feed or thermally demanding food-processing conditions may benefit from stability-oriented redesign, whereas enzymes already sufficiently stable under their intended process may require greater attention to matrix compatibility, substrate specificity, production yield, or degradation-product safety [79].

Multi-objective optimization should therefore be guided by application requirements rather than by maximizing the number of engineered properties. The practical objective is to identify a balanced performance profile that satisfies the intended use conditions without introducing unnecessary engineering complexity.

### 5.4. Integrated Design–Build–Test–Learn Workflows

The practical value of computational design becomes greatest when prediction and experimentation are connected through iterative Design–Build–Test–Learn workflows [20]. Rather than treating computational analysis, mutation construction, biochemical testing, and model refinement as independent stages, DBTL provides an organizational framework in which evidence generated during one cycle informs the objectives of the next.

During the Design stage, the target mycotoxin, intended application, and dominant engineering bottleneck are defined, and structural or sequence-based information is used to prioritize candidate interventions. The Build stage generates selected enzyme variants or production constructs using an application-appropriate expression system. During Test, variants are evaluated not only for catalytic activity but also for the properties relevant to their intended use, such as stability, expression, matrix performance, substrate selectivity, or degradation-product profiles. The Learn stage integrates these results to identify successful engineering principles, failed assumptions, and new bottlenecks that should guide the next design cycle.

For mycotoxin-detoxifying enzymes, an important feature of DBTL is the progressive increase in experimental realism. Early cycles may use purified enzymes and simplified substrates to establish mechanistic relationships, whereas later cycles should incorporate model matrices, authentic food or feed materials, processing-relevant conditions, and, where appropriate, pilot-scale validation. This progression prevents optimization against laboratory conditions that do not reflect the intended deployment environment.

DBTL should therefore not be interpreted as an obligation to automate every stage of enzyme development. Manual or partially automated workflows can follow the same iterative logic. Automation becomes advantageous when large libraries, standardized assays, and sufficiently informative datasets justify high-throughput experimentation [20]. Where data are sparse, smaller hypothesis-driven cycles may provide a more efficient development strategy.

**Figure 3:**
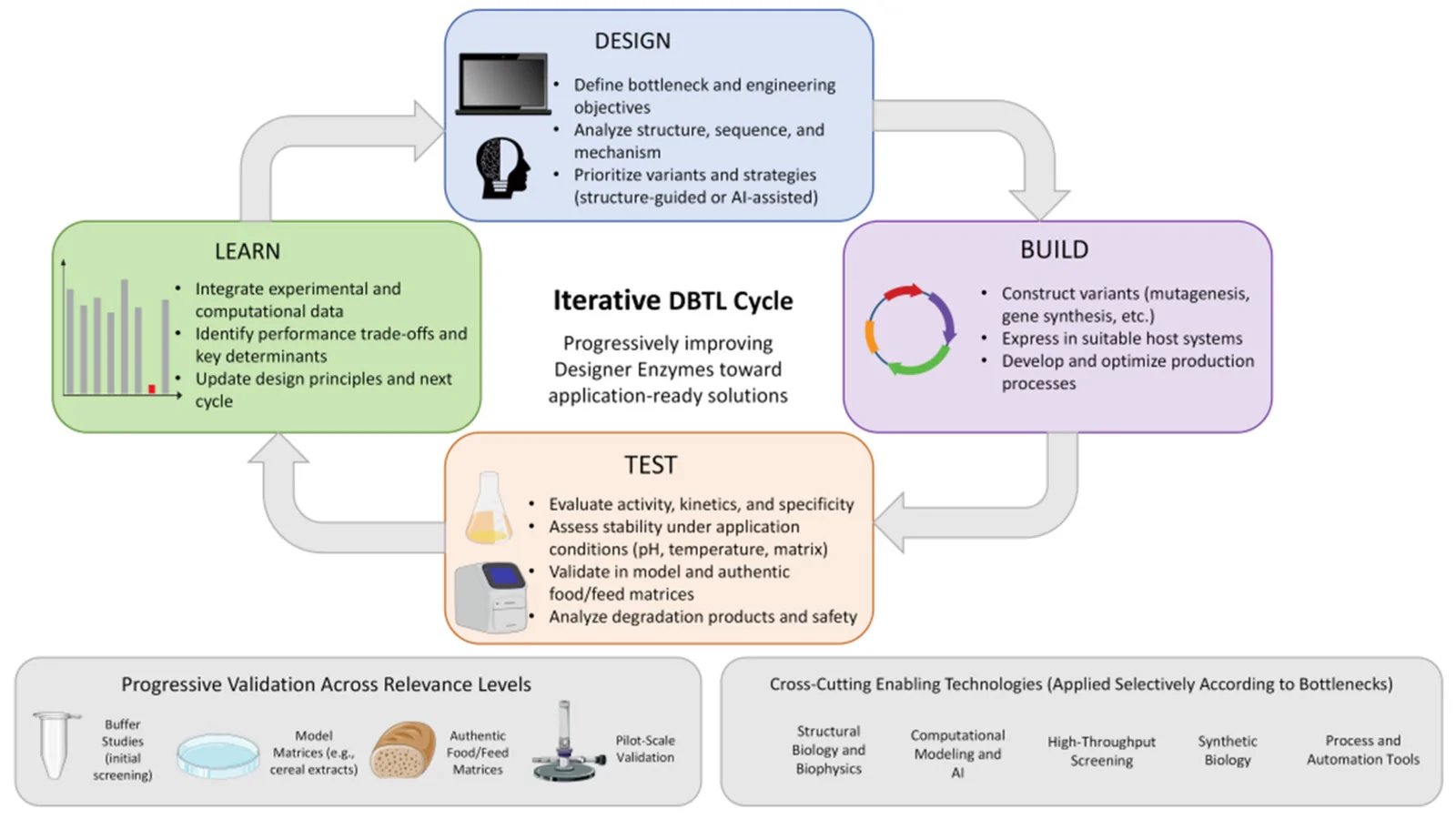
Iterative Design–Build–Test–Learn Workflow for Designer Enzyme Development. In the Design stage, target mycotoxins, application requirements, and dominant engineering bottlenecks are defined and candidate variants are prioritized using mechanistic, structural, or computational information. In the Build stage, selected variants are constructed and expressed using application-appropriate production systems. The Test stage evaluates catalytic performance, stability, matrix compatibility, and degradation-product safety under progressively more realistic conditions. In the Learn stage, experimental and computational data are integrated to identify performance trade-offs and refine the next design cycle. Validation progressively advances from buffer-based assays to model matrices, authentic food or feed systems, and pilot-scale conditions, while enabling technologies are applied selectively according to the identified bottleneck.

Within the Designer Enzyme concept, DBTL functions as an iterative decision framework that connects predictive design with experimental evidence. Its principal benefit is not technological complexity but the capacity to redirect engineering effort when experimental results reveal that the original bottleneck has changed. This iterative DBTL workflow, along with the progressive validation across relevance levels and the selective application of enabling technologies, is illustrated in Figure 3.

### 5.5. From Predictive Design to Deployment-Oriented Engineering

Computational biology and AI can substantially improve the efficiency of enzyme engineering, but they address only part of the translation pathway. Structural prediction, mutation prioritization, stability engineering, and DBTL primarily improve the ability to identify and optimize promising molecular variants. They do not, by themselves, resolve challenges associated with scalable production, formulation, multi-enzyme integration, food- or feed-specific delivery, and regulatory implementation.

Accordingly, computational design should be integrated selectively with downstream system engineering. Once the dominant molecular limitations have been sufficiently addressed, further progress may depend more strongly on production chassis, fermentation performance, formulation, modular enzyme systems, and application-specific deployment. These system-level strategies are discussed in the following section.

## 6. Synthetic Biology, Multi-Enzyme Systems, and Scalable Production

The computational and protein-engineering strategies discussed in Section 5 primarily address molecular-level limitations, but successful mycotoxin detoxification also requires translation of optimized enzymes into manufacturable and application-compatible systems. In practice, an enzyme with high catalytic activity may remain unsuitable for deployment if it cannot be produced economically, formulated stably, combined with complementary catalytic functions, or delivered effectively under the intended food or feed conditions [34,65].

Synthetic biology and bioprocess engineering provide complementary approaches for addressing these system-level constraints. Their contribution extends from host and secretion engineering to multi-enzyme assembly, cell-free catalysis, fermentation scale-up, immobilization, encapsulation, and process integration [34,99]. Importantly, these technologies should not be interpreted as obligatory components of every Designer Enzyme program. Their relevance depends on the specific bottleneck identified during development.

This section therefore focuses on how molecularly optimized detoxification enzymes can be converted into practical biocatalytic systems. Particular attention is given to scalable production, multi-enzyme detoxification, modular cell-free platforms, formulation technologies, and existing evidence of pilot-scale or commercial translation. These aspects primarily strengthen the Deployment Readiness dimension of the framework introduced in Section 3.

### 6.1. Chassis Engineering and Scalable Enzyme Production

Scalable and economically viable enzyme production is a prerequisite for practical mycotoxin-detoxification technologies. Expression systems should therefore be evaluated not only according to laboratory protein yield but also according to secretion efficiency, folding quality, fermentation robustness, downstream-processing requirements, production consistency, and regulatory suitability [100]. The optimal production host depends on the biochemical properties of the target enzyme and the intended application.

Bacterial expression systems such as *Escherichia coli* provide rapid growth, well-established genetic tools, and relatively straightforward process development for enzymes that do not require complex post-translational modifications [101]. However, intracellular expression can increase downstream recovery requirements, while inclusion-body formation or improper folding may limit the production of certain enzymes [102,103]. Secretion-capable bacterial hosts may reduce recovery costs when extracellular enzyme production can be achieved efficiently [104,105].

Yeast systems provide an alternative for proteins requiring more complex folding or when extracellular secretion is advantageous. Yeast-based production can facilitate recovery of secreted enzymes and offers established fermentation platforms for industrial biotechnology [99]. Other established industrial hosts, including filamentous fungi, may be advantageous for selected enzymes depending on secretion and processing requirements. Consequently, host selection should be based on enzyme-specific requirements rather than on the assumption that a single chassis is universally optimal.

Synthetic-biology-based production optimization can further involve promoter selection, gene-copy adjustment, codon optimization, secretion-signal engineering, chaperone co-expression, metabolic-burden reduction, and fermentation control. These strategies are particularly relevant when low expression yield or inconsistent production, rather than catalytic performance, constitutes the dominant translational bottleneck.

Importantly, scalable production should be considered during enzyme development rather than after molecular optimization has been completed. A variant that exhibits superior activity but requires substantially more complex production or purification may ultimately be less suitable than a slightly less active enzyme that can be manufactured consistently and economically. Within the Designer Enzyme framework, manufacturability therefore represents an integral component of deployment-oriented optimization.

### 6.2. Multi-Enzyme Systems for Co-Contamination and Masked Mycotoxins

Co-contamination is common in agricultural commodities, and a single highly specific detoxification enzyme may therefore be insufficient for practical risk reduction [106]. Representative contamination patterns include the co-occurrence of AFB1 and fumonisins in maize [107], DON and ZEN in maize and wheat, and more complex combinations involving multiple parent and modified toxins. Multi-enzyme systems provide one strategy for extending catalytic coverage across these heterogeneous contamination profiles.

The simplest configuration is an enzyme cocktail in which independently produced enzymes are combined before application. This strategy provides considerable flexibility because the proportion of each enzyme can be adjusted according to the contamination profile. It also allows individual components to be manufactured and optimized separately. However, different enzymes may possess incompatible pH optima, temperature profiles, formulation requirements, or storage stability, making simultaneous application challenging.

Fusion proteins provide an alternative architecture in which two or more catalytic domains are incorporated into a single polypeptide [62,63]. Such designs can simplify dosing and potentially improve proximity between sequential reactions, but they may also introduce folding constraints, altered domain mobility, or reduced activity of individual catalytic components. Consequently, fusion should be adopted only when the combined architecture provides a clear functional or manufacturing advantage.

More generally, scaffold-assisted or modular enzyme assemblies represent potential architectures for spatial organization of sequential catalytic steps, although direct application to mycotoxin detoxification remains comparatively limited. Catalytic components can be spatially organized while retaining greater independence than in a covalent fusion protein. Such arrangements may be particularly useful for sequential pathways in which the product of one reaction becomes the substrate of the next. Nevertheless, additional structural complexity must be justified by improved catalytic efficiency, reaction coordination, or process compatibility.

Masked mycotoxins present a particularly relevant case for sequential catalysis [108]. For glucoside-conjugated forms, a sequential strategy could use a deconjugating enzyme such as β-glucosidase to release the parent toxin, followed immediately by a second enzyme that detoxifies the exposed toxicophore. Such systems differ fundamentally from simple multi-toxin cocktails because reaction order and compatibility between individual catalytic steps become important. The intermediate released during de-masking must also be prevented from accumulating if it retains toxicological activity.

Accordingly, the design of multi-enzyme detoxification systems should consider substrate combinations, reaction sequence, enzyme compatibility, relative catalytic rates, matrix effects, product safety, manufacturing complexity, and cost. Increasing the number of catalytic components does not automatically improve deployment readiness. A multi-enzyme system is justified only when it addresses a contamination pattern that cannot be managed adequately by a simpler intervention.

Within the Designer Enzyme concept, multi-enzyme systems should therefore be regarded as application-specific solutions rather than defining characteristics of advanced enzyme engineering. Their value lies in expanding functional coverage where real contamination scenarios require coordinated detoxification of multiple or masked mycotoxins.

### 6.3. Cell-Free and Modular Biocatalytic Systems

Cell-free biocatalytic systems provide an alternative to detoxification strategies based on living microorganisms [23,51]. In such systems, detoxification is performed by isolated or formulated enzyme preparations rather than by viable production organisms, and multiple catalytic components can be combined where required. This feature may be particularly attractive for food-processing scenarios in which direct application of living engineered microorganisms would create additional process-control or regulatory considerations.

A major advantage of cell-free systems is modularity. Individual enzymes can be produced and characterized separately and, where appropriate, combined into defined preparations targeting multiple toxins [51]. Components can also be replaced or reformulated without redesigning an entire microbial pathway. This flexibility is particularly useful for multi-enzyme detoxification, where different toxins may require different catalytic activities or where contamination profiles vary among commodities.

However, removal of living cells does not eliminate the need for safety and manufacturing evaluation. Cell-free preparations still require control of enzyme purity, production-host-derived impurities, formulation components, activity stability, batch-to-batch consistency, degradation products, and intended-use exposure [23]. Their economic feasibility also depends on production yield, downstream recovery, enzyme dose, and process integration.

Cell-free platforms should therefore be viewed as modular deployment formats rather than inherently safer or simpler solutions in all cases. Their suitability depends on whether modularity, controllability, and separation of production from application provide clear advantages for the intended food or feed process.

### 6.4. Formulation, Immobilization, and Encapsulation

Formulation provides the physical interface between an engineered enzyme and its intended application environment. Even when an enzyme possesses suitable intrinsic catalytic properties, exposure to heat, moisture, proteases, matrix inhibitors, or prolonged storage can substantially reduce effective activity [48,49]. Encapsulation, immobilization, protective coatings, carrier-based formulations, and controlled-release systems therefore provide additional strategies for improving functional stability without requiring further modification of the enzyme sequence [51,89,109].

For feed applications, formulation often needs to address two sequential challenges: survival during feed manufacture and functional release after ingestion. Protective coating or encapsulation can be used to improve enzyme survival during processing and subsequent gastrointestinal exposure [51]. More generally, enteric or pH-responsive delivery systems may be considered when release at a specific gastrointestinal location is required. Immobilization or carrier-assisted delivery may improve enzyme stability, reusability, or local retention, although the magnitude of these benefits is formulation- and enzyme-specific.

Food applications impose different formulation requirements. Depending on the processing step, the principal objective may be protection against moderate heating, stabilization in an aqueous or low-moisture matrix, reduction in non-specific adsorption, or facilitation of enzyme removal or inactivation after detoxification. Because food applications must also preserve sensory and technological quality, formulation components should be compatible with the product and should not introduce undesirable changes in texture, flavor, color, or processing behavior.

Immobilization provides additional advantages in selected processing environments by facilitating enzyme recovery, repeated use, or spatial control of catalysis. However, immobilization may also reduce substrate accessibility or introduce mass-transfer limitations, particularly in heterogeneous food or feed matrices. Consequently, an immobilized system should be evaluated under authentic process conditions rather than solely according to retained activity in buffer.

Formulation should therefore be optimized according to a clearly defined application rather than treated as a universal downstream solution. In some cases, sequence-level stability engineering may provide the simplest route to improved performance; in others, formulation may achieve the required robustness without additional molecular redesign. The appropriate choice depends on technical performance, process compatibility, manufacturing cost, and regulatory acceptability.

### 6.5. Existing Pilot-Scale and Commercial Translation

Although many reported mycotoxin-detoxifying enzymes remain at laboratory or pre-deployment stages, selected technologies have progressed into application-oriented development, formal regulatory evaluation, and authorized feed use [51]. These examples are important because they reveal which requirements become critical once enzyme development moves beyond purified biochemical assays.

Fumonisin esterase systems provide the clearest current example of this transition. FumD-based detoxification has progressed beyond enzyme characterization to efficacy assessment in contaminated feed, toxicological evaluation of hydrolyzed fumonisin products, target-animal studies, regulatory review, and authorized feed-additive application [33]. This trajectory demonstrates that industrial translation requires integration of catalytic efficacy with manufacturing consistency, formulation, safety, intended-use conditions, and regulatory documentation rather than optimization of enzyme activity alone.

Other mycotoxin-detoxifying systems remain at different points along this translation pathway. ZEN hydrolases have accumulated substantial structural, engineering, and application-oriented evidence, although publicly available pilot-scale, regulatory, and commercial deployment data remain comparatively limited. Fhb7 is supported strongly by mechanistic and biological evidence but has been studied primarily in the context of plant resistance rather than as an industrial cell-free enzyme preparation [35]. AFB1-transforming enzymes and OTA hydrolases have increasingly progressed from catalytic characterization toward immobilization, formulation, and application-oriented engineering [109], but systematic large-scale validation, cross-matrix reproducibility, and product-safety evidence remain less extensive than for the most mature fumonisin esterase systems.

Pilot-scale evaluation should therefore bridge the gap between laboratory optimization and commercial implementation. Relevant endpoints include fermentation reproducibility, recovery and formulation of the enzyme preparation, storage stability, performance across independent batches of authentic commodities, activity under realistic processing conditions, dose–response behavior, product-quality effects, degradation-product profiles, and preliminary economic feasibility. The specific endpoints differ between food and feed applications and should be defined according to the intended use scenario.

Importantly, lack of published pilot-scale evidence should not automatically be interpreted as technical failure. Industrial process details may also be incompletely represented in peer-reviewed literature because some development information remains proprietary or is reported primarily through regulatory dossiers rather than research articles. Nevertheless, from the perspective of scientific assessment, absence of accessible scale-up evidence limits the strength with which deployment readiness can be evaluated.

Existing commercial and regulatory examples therefore serve as empirical benchmarks for Designer Enzyme development. They demonstrate that the transition from enzyme discovery to practical application is not a single engineering step but a sequence involving production, formulation, realistic-matrix testing, toxicological verification, scale-up, and regulatory acceptance.

### 6.6. Section Summary: From Molecular Optimization to Deployable Systems

The transition from an engineered enzyme to a deployable mycotoxin-detoxification system depends on more than molecular performance. Scalable production determines whether sufficient enzyme can be manufactured consistently; multi-enzyme architectures address contamination scenarios that exceed the substrate range of individual catalysts; cell-free systems provide modular deployment options; and formulation determines whether catalytic activity can be preserved under real processing and physiological conditions. Existing commercial examples further demonstrate that efficacy, safety, manufacturing, and regulatory evidence must ultimately converge before practical implementation is achieved.

These requirements are strongly application-dependent. Food-oriented systems must prioritize compatibility with processing operations, food matrices, product quality, and consumer safety, whereas feed-oriented systems additionally face feed-manufacturing and gastrointestinal constraints. The following section therefore moves from the engineering platform itself to the broader food- and feed-specific requirements governing deployment, sustainability, and regulatory translation.

## 7. From Laboratory Innovation to Food- and Feed-Specific Deployment

The engineering strategies discussed in Section 5 and Section 6 address molecular optimization, scalable production, multi-enzyme integration, and formulation, but successful translation ultimately depends on the conditions under which the resulting biocatalytic system is used. Food and feed applications impose distinct processing, physiological, quality, safety, and regulatory requirements and should therefore not be treated as interchangeable deployment scenarios [23].

For food applications, deployment is primarily determined by compatibility with specific processing operations, authentic food matrices, product quality, degradation-product safety, and the regulatory status of the enzyme preparation and its production system. Feed applications additionally require consideration of conditioning, pelleting, storage, gastrointestinal exposure, target-animal efficacy, and feed-additive regulatory requirements. These differences directly affect how Deployment Readiness and Safety and Regulatory Preparedness should be interpreted within the framework proposed in Section 3.

This section therefore distinguishes food- and feed-specific deployment requirements before considering the broader implications of enzyme-based mycotoxin detoxification for sustainability, food-loss reduction, regulation, and future technological development.

### 7.1. Food-Specific Deployment Requirements

Food applications require detoxification enzymes to function within defined processing operations while preserving the nutritional, sensory, and technological properties of the product [47,87]. Relevant application stages may include grain treatment, soaking, milling-associated processing, fermentation, brewing, beverage processing, or other operations in which temperature, pH, water availability, and matrix composition differ substantially. Consequently, evidence obtained in purified buffer should be supplemented by validation in the specific commodity and process for which the enzyme is intended [110].

Matrix compatibility is particularly important because food components can alter both enzyme performance and toxin accessibility. Food-matrix components may alter enzyme activity, toxin accessibility, or mass transfer through effects such as non-specific interactions, competing substrates, or restricted diffusion [47]. Processing temperature and pH further influence enzyme stability and reaction rate. Therefore, a food-oriented enzyme should be evaluated under realistic combinations of matrix composition, processing conditions, enzyme dose, and reaction time rather than through isolated biochemical parameters alone.

Food deployment also introduces product-quality considerations that are less prominent in many feed applications. An effective detoxification process should not produce unacceptable changes in color, flavor, texture, nutritional composition, or functional properties of the food [87,111]. This requirement is especially relevant for broad-specificity oxidative enzymes, which may react with non-target matrix components in addition to the intended mycotoxin. Accordingly, evaluation of food-oriented Designer Enzymes should include both toxin reduction and preservation of relevant quality attributes.

The timing of enzyme addition must also be integrated with process design. If the enzyme is exposed to a subsequent severe thermal step, either sufficient thermostability or an appropriate protective formulation may be required. Alternatively, enzyme application may be positioned after heating or during a processing stage that provides more favorable catalytic conditions. Process integration can therefore reduce the need for unnecessary molecular engineering when an effective intervention point can be selected operationally.

Finally, food applications require clear characterization of transformation products and the safety of the enzyme preparation itself. Because direct toxin disappearance does not demonstrate detoxification, analytical identification of major products should be combined with biological safety evidence appropriate to the intended exposure. Production-organism safety, residual process materials, enzyme inactivation or removal where relevant, and compliance with the applicable food-enzyme regulatory pathway should also be considered before commercialization.

Food-specific deployment readiness should therefore be defined by demonstrated performance in the intended food matrix and process, preservation of product quality, verified reduction in toxicological risk, and compatibility with the relevant manufacturing and regulatory environment.

### 7.2. Feed-Specific Deployment Requirements

Feed applications present a distinct deployment pathway because detoxification enzymes may encounter processing stress before ingestion and physiological stress after ingestion [48,49]. Depending on the intended mode of action, an enzyme may be expected to detoxify mycotoxins within the feed matrix before consumption, remain active during gastrointestinal transit, or contribute to toxin transformation at a defined site within the digestive tract. These different use scenarios require different combinations of stability, formulation, and efficacy evidence.

During feed manufacture, conditioning and pelleting in particular can expose enzymes to elevated temperature and moisture, while subsequent handling and storage introduce additional stability requirements [11]. These factors can reduce catalytic activity before the feed reaches the animal. Accordingly, feed-oriented enzyme development may require thermostability engineering, protective formulation, post-pelleting application, or other process adaptations depending on the manufacturing system.

After ingestion, enzymes intended to remain functional encounter a highly dynamic gastrointestinal environment. Gastric acidity can destabilize enzyme structure or reduce catalytic activity, while pepsin, trypsin, and other proteases may degrade the protein before detoxification is complete [49]. Bile salts, intestinal enzymes, microbial activity, and dietary components may further influence enzyme stability and catalytic performance. Therefore, gastrointestinal robustness should be evaluated when in vivo activity forms part of the intended mode of action.

Formulation provides one strategy for addressing these sequential challenges. Encapsulation, protective coatings, immobilization, or carrier systems can be used to improve enzyme stability or delivery, whereas enteric or pH-responsive release may be considered when site-specific gastrointestinal activity is required [51]. However, formulation should be selected according to the intended site and timing of detoxification rather than applied routinely to all feed enzymes. An enzyme designed primarily to act in stored or processed feed may not require the same gastrointestinal protection as one intended for intestinal activity.

Target-animal evidence represents an additional component of feed deployment. Analytical reduction of a mycotoxin in feed does not necessarily establish biological efficacy after ingestion [24]. Depending on the intended claim, feeding studies may therefore be required to evaluate exposure biomarkers, animal tolerance, performance, intestinal health, or other toxicologically relevant endpoints. Degradation products should likewise be characterized to ensure that enzymatic transformation results in genuine risk reduction rather than simple chemical modification.

Feed-specific Deployment Readiness should therefore integrate manufacturing stability, storage performance, authentic-feed efficacy, gastrointestinal functionality where relevant, target-animal outcomes, degradation-product safety, and regulatory requirements for feed additives. These criteria differ fundamentally from those governing direct food-processing applications and should be evaluated separately. The principal gastrointestinal constraints and corresponding engineering responses are summarized in Section 2.3.

### 7.3. Sustainability and Food-Loss Reduction

Mycotoxin contamination contributes to food and feed losses through multiple pathways, including rejection of contaminated commodities, downgrading from food to feed use, reduced livestock productivity, monitoring and control costs, and restrictions on domestic or international trade [112,113]. The historically cited estimate that approximately 25% of global food crops are affected by mycotoxins has been widely discussed and subsequently re-evaluated, with more recent evidence indicating that the prevalence of detectable contamination depends strongly on commodity, geographical region, analytical sensitivity, and the threshold used to define contamination [1].

From a resource-use perspective, commodities that are rejected or downgraded because of mycotoxin contamination embody previously invested land, water, fertilizer, energy, labor, storage, and transportation resources. Effective detoxification could therefore provide benefits beyond chemical risk reduction if contaminated commodities can be safely recovered for their intended use rather than discarded. However, this potential should not be assumed automatically, because recovery is only beneficial when detoxification efficacy, product safety, processing quality, and economic feasibility are all demonstrated.

Enzymatic detoxification may offer sustainability advantages because many enzyme-catalyzed reactions operate under comparatively mild conditions and can provide greater chemical specificity than broad physical or chemical treatments. Nevertheless, the environmental advantage of an enzymatic process depends on enzyme manufacturing, formulation, required dose, process energy, treatment duration, recovery strategy, and the extent to which the intervention prevents commodity loss [114]. Therefore, lower reaction severity alone should not be interpreted as proof of superior life-cycle performance.

A more rigorous assessment of sustainability should integrate detoxification efficiency with food- or feed-recovery rate, energy consumption, water use, enzyme-production burden, formulation inputs, waste generation, and techno-economic performance. Life-cycle assessment and techno-economic analysis would be particularly useful for comparing Designer Enzyme-based interventions with sorting, adsorption, chemical detoxification, or commodity disposal under equivalent contamination scenarios.

The sustainability contribution of Designer Enzymes should therefore be evaluated according to measurable system-level outcomes rather than catalytic performance alone. Relevant indicators may include the amount of commodity retained in the food or feed chain, reduction in waste generation, resource-use efficiency, processing cost, and life-cycle environmental impact. This broader perspective links mycotoxin detoxification to food-security and resource-efficiency objectives without assuming benefits that have not yet been quantitatively demonstrated.

### 7.4. Regulatory Readiness and Global Governance

Regulatory readiness is a major determinant of whether mycotoxin-detoxifying enzymes can progress from technical feasibility to commercial application. Importantly, regulatory pathways differ not only among jurisdictions but also according to whether the enzyme is intended for food processing, feed use, or another technological function [115]. Consequently, a single generic regulatory pathway cannot be assumed for all Designer Enzyme applications.

For food applications, regulatory assessment may consider the safety of the production organism, manufacturing process, enzyme preparation, residual materials, intended technological function, dietary exposure, and the toxicological properties of reaction products [116]. Where an enzyme is introduced directly into food processing, additional consideration may be required regarding whether the enzyme remains active or present in the final product and whether the detoxification reaction affects food composition or quality.

Feed applications are evaluated within a different regulatory context. Depending on jurisdiction and intended claim, evidence may include production-strain safety, manufacturing consistency, target-animal tolerance, efficacy in contaminated feed, degradation-product characterization, consumer safety relating to animal-derived foods, worker safety, and environmental risk. Fumonisin esterase systems provide an important example in which successful translation required evidence extending from enzymatic efficacy to animal and regulatory evaluation rather than biochemical activity alone.

Regulatory requirements also vary geographically. The European Union applies structured safety and efficacy assessment to feed additives and food-related enzyme applications under relevant regulatory frameworks [117]. The United States follows application- and product-specific pathways [118], China regulates feed additives under its national feed and feed-additive regulatory framework and maintains specific standards for feed enzyme preparations and individual enzyme categories [119]. In Japan, substances used during food manufacture, including processing aids under the Japanese definition, are regulated within the Food Sanitation Act framework, with permitted additives subject to the applicable designation and use requirements [120].

Degradation-product safety is particularly important for detoxification enzymes because reduction of the parent mycotoxin does not necessarily demonstrate reduction in biological risk. Regulatory and safety assessment may therefore require characterization of relevant reaction products and, where exposure is plausible, evidence supporting their toxicological acceptability. This consideration is especially important for oxidative reactions that may generate multiple or incompletely characterized intermediates.

Regulatory preparedness should consequently begin during development rather than after technical optimization has been completed. Early definition of the intended food or feed application can determine which safety studies, manufacturing controls, efficacy endpoints, and product-characterization data will ultimately be required. Within the Designer Enzyme concept, regulatory-by-design therefore refers to aligning the engineering and evidence-generation pathway with the intended regulatory context from the outset, rather than attempting to retrofit an optimized enzyme into an unsuitable approval pathway. A comparative overview of application-specific regulatory considerations across key jurisdictions and use contexts is provided in Table 9.

### 7.5. Future Perspectives and Next-Generation Designer Enzyme Ecosystems

Future development of mycotoxin-detoxifying enzymes is likely to shift from isolated optimization of catalytic properties toward coordinated engineering across molecular, process, safety, and deployment levels. However, the most immediate priorities are comparatively practical: standardized testing, authentic-matrix validation, improved reporting of degradation products, scalable production, and clearer distinction between food- and feed-specific performance requirements [16]. Strengthening these foundations is essential before more advanced autonomous engineering concepts can provide substantial value.

In the near term, greater standardization is needed across enzyme assays, toxin concentrations, matrices, processing conditions, activity measurements, and reporting practices. Current variability limits cross-study comparison and complicates interpretation of whether apparent improvements reflect true engineering advances or differences in experimental design. Shared benchmarking protocols and open datasets integrating sequence, structure, kinetics, stability, mutation outcomes, matrix performance, and toxicological evidence would therefore strengthen both conventional and AI-assisted enzyme development.

As higher-quality datasets become available, computational and automated DBTL approaches may increasingly support multi-objective engineering [122,123]. Machine-learning-assisted mutation prioritization, automated screening, and iterative experimental feedback could improve the efficiency of exploring trade-offs among activity, stability, selectivity, expression, and other application-relevant properties. The value of such automation will depend on dataset quality and assay relevance.

Multi-enzyme and modular biocatalytic systems are also likely to become increasingly important for co-contamination and masked mycotoxins [51]. Their development should focus on compatibility among catalytic components, control of reaction sequence, product safety, formulation, and economic feasibility rather than maximizing system complexity. Modular architectures could be particularly useful when contamination profiles vary across commodities or regions because individual catalytic components could, in principle, be adjusted without redesigning the entire platform.

At a longer time scale, digital-twin and data-driven process models may provide opportunities to connect enzyme behavior with food and feed manufacturing conditions, contamination profiles, formulation performance, and process economics [124]. Such tools could eventually support prediction of where and when an enzymatic intervention would provide the greatest benefit within a supply chain. However, their application to mycotoxin-detoxifying enzyme deployment would require standardized experimental data and validated process models that are not yet widely available.

Future evaluation should also integrate sustainability and regulatory considerations earlier in development. Life-cycle analysis, techno-economic assessment, product-recovery metrics, toxicological evidence, and jurisdiction-specific regulatory requirements can help determine whether an apparently successful enzyme actually provides a practical advantage over existing mitigation strategies. This broader evidence base will be necessary if Designer Enzymes are to progress from laboratory innovations into credible food- and feed-management technologies.

**Figure 4:**
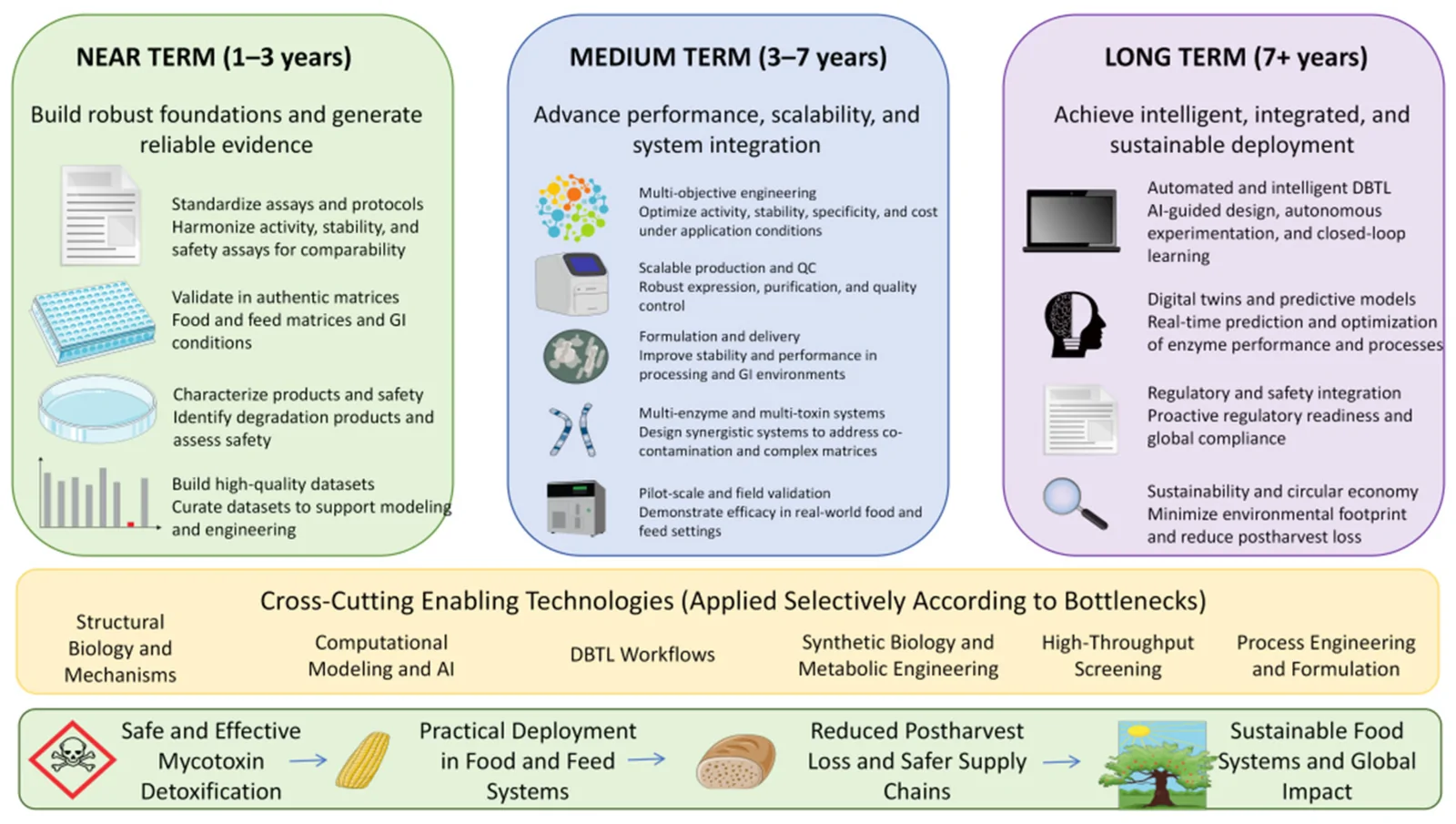
Roadmap Toward Application-Ready and Sustainable Designer Enzyme Platforms for Mycotoxin Detoxification.

Overall, the next stage of the field should not be defined simply by the increasing use of artificial intelligence, synthetic biology, or automation. Progress will depend on whether these tools resolve clearly identified translational bottlenecks. The central challenge is therefore to connect mechanistic knowledge and predictive engineering with scalable production, authentic-matrix performance, verified toxicological safety, food- or feed-specific deployment, and regulatory acceptance. In this sense, the future of Designer Enzymes lies in more disciplined integration rather than technological complexity alone. This phased roadmap—from near-term foundational standardization to medium-term system integration and long-term intelligent deployment—is presented in Figure 4.

These considerations provide the basis for the concluding assessment of how the Designer Enzyme concept can contribute to the translation of mycotoxin-detoxifying biocatalysts from laboratory research to practical food and feed applications.

## 8. Conclusions

Mycotoxin-detoxifying enzymes have advanced substantially from initial activity screening toward increasingly sophisticated mechanism-guided engineering, matrix validation, and application-oriented development. However, the literature reviewed here demonstrates that catalytic efficiency alone remains an insufficient indicator of practical value. Successful translation depends on whether an enzyme can maintain function under its intended food or feed conditions while also satisfying requirements related to production, formulation, degradation-product safety, regulatory acceptance, and process compatibility.

The available evidence also shows that different mycotoxin-enzyme systems follow markedly different translational trajectories. ZEN lactonases possess a comparatively strong mechanistic and engineering foundation, whereas Fhb7 remains more closely associated with biological resistance and mechanistic discovery. AFB1-transforming laccases provide broad catalytic potential but require particularly careful evaluation of reaction selectivity and degradation products. OTA hydrolases have benefited from increasingly detailed structural and mechanistic characterization, while fumonisin esterases represent one of the clearest examples of progression toward regulated feed application. These differences demonstrate that technological maturity cannot be adequately represented by catalytic performance or computational sophistication alone.

To organize these heterogeneous development pathways, this review uses the Designer Enzyme concept as a literature-derived, deployment-oriented framework encompassing mechanistic understanding, predictive design integration, multi-objective optimization, deployment readiness, and safety and regulatory preparedness. The framework is intended primarily as a diagnostic tool rather than as a formally validated scoring system. Its value lies in identifying the dominant bottleneck for a particular enzyme–mycotoxin–application combination and in guiding the selection of the next engineering or validation step.

A central conclusion of this review is that food and feed applications must be evaluated separately. Food-oriented enzymes require process-specific matrix compatibility, preservation of product quality, appropriate reaction-product safety, and alignment with food-processing regulations. Feed-oriented enzymes may additionally require stability during conditioning and pelleting, gastrointestinal functionality, target-animal efficacy, and compliance with feed-additive requirements. Treating these application environments as interchangeable can obscure the actual factors limiting deployment.

The evidence further indicates that future progress should not be defined simply by the increasing use of artificial intelligence, synthetic biology, automation, or multi-enzyme systems. These technologies are most valuable when they address a clearly identified translational limitation. Structure-guided and AI-assisted design can reduce molecular search space; DBTL workflows can improve iterative learning; synthetic biology and host engineering can support manufacturability; formulation can improve application stability; and modular or multi-enzyme systems can extend detoxification to co-contaminated or masked mycotoxin scenarios. Their contribution should therefore be judged by whether they improve real deployment outcomes rather than by technological complexity itself.

From a broader food-system perspective, enzymatic detoxification may contribute to reduced commodity loss and more efficient resource use when contaminated materials can be safely retained within food or feed chains. However, these benefits require direct evidence from realistic processing, toxicological, economic, and life-cycle assessments rather than assumptions based solely on mild reaction conditions or toxin disappearance. Regulatory and sustainability considerations should therefore be incorporated earlier in development, alongside catalytic and manufacturing objectives.

Overall, the field is moving from enzyme-centered optimization toward application-defined biocatalyst development. The most important next step is not simply to discover more mycotoxin-transforming enzymes, but to generate stronger and more standardized evidence linking molecular mechanism, engineering performance, authentic-matrix efficacy, scalable production, safety, and regulatory feasibility. Within this context, the Designer Enzyme concept provides a practical framework for organizing existing knowledge and directing future development toward biocatalysts that are not only active in the laboratory but also credible for real food and feed applications.

## Figures and Tables

**Table 1:** Comparative advantages, limitations, implementation challenges, and potential consequences of major mycotoxin mitigation strategies.

Strategy	Main Advantages	Main Limitations	Implementation Challenges	Potential Consequences of Application	References
Physical treatment	Rapid implementation; applicable to bulk commodities; does not require biological production systems; some approaches can reduce contamination before processing	Frequently incomplete toxin removal; may redistribute rather than destroy toxins; effectiveness depends strongly on commodity and contamination pattern	Separation efficiency, equipment requirements, energy input, scalability, and contamination heterogeneity	Loss of edible material, reduced commodity yield, possible nutrient loss, and residual contamination	[12,13,14]
Chemical treatment	Can achieve substantial toxin conversion under selected conditions; potentially rapid reaction kinetics; applicable to heavily contaminated materials	Risk of chemical residues or secondary reaction products; possible damage to nutrients and sensory quality; limited consumer acceptance in some food applications	Process control, reagent handling, waste treatment, corrosion, safety management, and regulatory restrictions	Reduced parent-toxin concentration but possible formation of undesirable by-products, altered food/feed quality, and additional chemical burden	[12,15]
Natural enzymatic treatment	High catalytic specificity; mild reaction conditions; lower risk of extensive matrix damage; direct modification of toxicity-associated structures	Limited thermostability, narrow substrate range, insufficient matrix tolerance, low production yield for some enzymes, and incomplete degradation-product characterization	Enzyme production, purification, activity retention during processing, substrate accessibility, formulation, and verification of product safety	Selective detoxification with comparatively limited changes to the surrounding matrix, but efficacy may decline under realistic food/feed conditions	[11,12,16]
Engineered/Designer Enzyme-based treatment	Potential for simultaneous optimization of activity, stability, specificity, expression, matrix tolerance, and deployment-related properties; can be tailored to defined food or feed applications	Greater development complexity; requires stronger mechanistic, manufacturing, safety, and regulatory evidence; engineering gains may involve trade-offs among properties	Multi-objective optimization, scale-up, formulation, authentic-matrix validation, degradation-product toxicology, and regulatory approval	Greater robustness and application specificity are possible if engineering and safety requirements are successfully integrated; however, increased development cost and evidence requirements may accompany higher system complexity	[11,16,17]
Strategy	Main advantages	Main limitations	Implementation challenges	Potential consequences of application	

**Table 2:** Major structural toxicophores, enzymatic detoxification targets, and representative enzyme systems for major mycotoxins.

Mycotoxin	Primary Toxicophore	Major Toxic Effects	Detoxification Strategy	Representative Enzymes	Representative References
AFB1	Difuran moiety, particularly the C8–C9 double bond, together with the lactone-containing coumarin system	Hepatotoxicity, genotoxicity, carcinogenicity	Oxidative, reductive, or hydrolytic modification of toxicity-associated structural regions	Laccases, MnPs, aflatoxin oxidases, reductases	[16,34]
DON	12,13-Epoxide-containing trichothecene core	Ribotoxicity, intestinal and immunotoxic effects	Epoxide modification, oxidation/epimerization, or other enzymatic transformations	Fhb7 GST, DepA, AKRs	[16,28,35]
ZEN	Resorcylic acid lactone ring	Estrogenicity and reproductive toxicity	Lactone-ring hydrolysis	ZHD101 and related ZEN lactonases	[26,27]
OTA	Amide bond linking the isocoumarin moiety to L-phenylalanine	Nephrotoxicity	Amide-bond hydrolysis	Carboxypeptidases, amidohydrolases	[30,31]
Fumonisins	Tricarballylic acid side chains and aminopolyol backbone associated with sphingolipid disruption	Disruption of sphingolipid metabolism	Ester hydrolysis of tricarballylic acid groups	FumD and related fumonisin esterases	[36,37]

**Table 3:** Food-specific matrix and processing barriers affecting detoxification enzyme performance.

Food-Related Factor	Potential Effect on Enzyme Performance	Engineering or Deployment Response	Representative References
Temperature	Thermal denaturation or loss of catalytic activity	Thermostability engineering, immobilization, or process-stage optimization	[11,44]
pH variation	Reduced catalytic activity or structural stability outside the enzyme-specific optimum	pH-profile optimization or selection of enzymes with broader pH tolerance	[45,46]
Matrix macromolecules	Altered toxin accessibility, non-specific interactions, or reduced apparent catalytic efficiency	Surface engineering, immobilization, or matrix-specific formulation	[47]
Lipid-rich matrices	Altered enzyme–toxin partitioning and substrate accessibility	Interface-oriented formulation or amphiphilicity engineering	[44]
Polyphenol-rich matrices	Potential interference with enzyme activity or substrate accessibility	Matrix-specific validation and, where necessary, engineering for improved tolerance	[46]
Brewing/fermentation conditions	Dynamic changes in pH, temperature, composition, and residence time	Process-specific enzyme selection and validation	[38,39]
Baking/thermal processing	Heat exposure may reduce enzyme activity before detoxification is completed	Thermostable variants, immobilization, or application at a more suitable processing stage	[11]
Product-quality requirements	Potential changes in sensory, nutritional, or technological properties must be excluded	Validation of color, flavor, texture, composition, and other relevant quality attributes	[45,46,47]

**Table 4:** Feed-specific gastrointestinal barriers and engineering strategies for mycotoxin-detoxifying enzymes.

Gastrointestinal Factor	Potential Impact	Engineering or Deployment Strategy	Representative References
Gastric acidity	Reduced activity or structural stability of acid-sensitive enzymes	Acid-tolerant engineering, protective formulation, or enteric delivery	[17,49]
Pepsin and gastric proteases	Proteolytic inactivation of susceptible enzymes	Encapsulation, protease-resistant engineering, or protective formulation	[49]
Pancreatic and intestinal proteases	Partial proteolysis and activity loss of susceptible enzymes	Protease-resistant redesign, immobilization, or encapsulation	[17]
Bile-associated intestinal conditions	Enzyme-specific changes in structural stability and catalytic activity	Stability screening and protective formulation	[55]
Gut microbiota and intestinal matrix	Modification of enzyme persistence, toxin accessibility, and apparent detoxification performance	Validation under simulated or in vivo gastrointestinal conditions	[56]
Dynamic gastrointestinal environment	Combined effects of pH, proteases, bile components, transit time, and feed matrix	Application-specific formulation and progressive gastrointestinal validation	[55]

**Table 5:** Comparison between conventional engineered enzymes and Designer Enzymes.

Feature	Conventional Enzyme Optimization: Common Emphasis	Deployment-Oriented Designer Enzyme Development
Initial objective	Improvement of one or more predefined biochemical or functional properties	Definition of intended-use requirements and translational constraints before optimization
Engineering target	Activity, stability, specificity, expression, or other selected enzyme properties	Application-dependent combination of catalytic, production, formulation, matrix, safety, and deployment traits
Design strategy	Rational design, directed evolution, screening, and increasingly computationally assisted engineering	Selective use of structure-guided design, predictive tools, DBTL, production engineering, or formulation according to the identified translational bottleneck
Validation strategy	Frequently begins with biochemical characterization under simplified laboratory conditions	Progressive validation from purified systems to model matrices, authentic food/feed systems, and intended-use conditions
Manufacturing considerations	May be incorporated during development, particularly in industrial enzyme programs	Explicitly incorporated when expression yield, production consistency, scale-up, or formulation represents a translational constraint
Safety assessment	Usually application-dependent and may occur as development progresses toward use	Degradation-product safety and intended-use toxicological evidence are treated as explicit deployment criteria
End point	Improved enzyme variant or biocatalyst with enhanced target properties	Biocatalytic system with explicitly characterized suitability and remaining limitations for a defined food or feed application

This comparison represents a conceptual distinction used in this review rather than a universal classification of conventional and Designer Enzyme development. Conventional industrial enzyme engineering may also incorporate manufacturing, formulation, safety, and application-related considerations. The distinction emphasized here is the explicit definition and integration of deployment requirements as organizing objectives from the early stages of development.

**Table 6:** Evidence dimensions for literature-based assessment of mycotoxin-detoxifying enzymes.

Dimension	Core Question	Early Evidence	Intermediate Evidence	Advanced Evidence	Reference
Mechanistic Understanding	Is the catalytic mechanism sufficiently characterized?	Detoxification activity observed, but the mechanism remains largely inferred	Key catalytic residues, transformation pathway, or major products partially validated	Reaction pathway, catalytic determinants, substrate interactions, and major transformation products experimentally characterized	[25]
Predictive Design Integration	Are engineering decisions guided by predictive information?	Predominantly empirical screening or unguided variant testing	Structural modeling, sequence analysis, or limited computation informs candidate selection	Iterative use of structural, computational, or AI-assisted prediction with experimental feedback to guide subsequent engineering	[19]
Multi-Objective Optimization	Are multiple deployment-relevant traits considered together?	Improvement focused primarily on a single property	Two or more properties optimized, with trade-offs recognized	Coordinated optimization of catalytic and application-relevant properties with explicit consideration of trade-offs	[70]
Deployment Readiness	Has the enzyme been evaluated under conditions relevant to intended use?	Predominantly buffer-based or simplified laboratory characterization	Validation in model systems or authentic food/feed matrices	Evidence from pilot-scale, industrially relevant, validated in vivo, or commercial-use conditions appropriate to the intended application	[23]
Safety and Regulatory Preparedness	Is detoxification safety supported beyond disappearance of the parent toxin?	Transformation products or biological consequences remain insufficiently characterized	Major products identified and preliminary biological safety assessed	Toxicological, manufacturing, production-organism, efficacy, and regulatory evidence sufficiently developed for the intended use	[23]

Evidence levels are qualitative descriptors synthesized from the literature and are not intended as universally validated numerical thresholds. Their interpretation should be adapted to the target mycotoxin, enzyme class, and specific food or feed application.

**Table 7:** Comparative evidence profiles and translational bottlenecks of representative mycotoxin-detoxifying enzyme systems.

Enzyme/System	Target Mycotoxin	Established Evidence	Current Translational Strength	Major Remaining Bottleneck	Representative References
ZHD101-related lactonases	ZEN	Defined lactone-hydrolysis mechanism; structural and engineering evidence; markedly reduced estrogenicity of major hydrolysis products; regulatory assessment available for selected ZEN hydrolases	Strong mechanistic and protein-engineering foundation with emerging deployment evidence	Industrial food/feed validation, formulation, manufacturing robustness, and broader application-specific regulatory translation	[26,27,78]
Fhb7-GST	DON/trichothecenes	Defined biological function and glutathione-dependent transformation of trichothecenes; reduced toxicity of major glutathione adducts	Strong mechanistic and toxicological platform	Stand-alone enzyme production, processing stability, formulation, authentic-matrix validation, and industrial development	[35,80]
CotA and related laccases	AFB1	Oxidative transformation; identified degradation products in selected systems; mutational and structure-guided engineering; broadened substrate scope in some laccases	Flexible oxidative platform with substantial engineering potential	Reaction selectivity, condition-specific product toxicology, systematic authentic food-matrix validation	[81,83]
OTA hydrolases/amidohydrolases	OTA	Defined amide-bond hydrolysis; structural and rational-engineering evidence; emerging validation in authentic food matrices	Strong and rapidly advancing mechanistic basis	Broader food-matrix validation, scalable production, formulation, and application-specific safety evidence	[30,89]
FumD/fumonisin esterases	Fumonisins	Defined ester hydrolysis; in vivo feeding and efficacy evidence; manufacturing and safety assessment; regulatory evaluation and authorized feed-additive use	Comparatively advanced feed deployment	Formulation refinement, processing robustness, broader feed-matrix validation, dose optimization, and jurisdiction-specific expansion	[24,88]

The evidence profiles represent a qualitative synthesis of the published literature and are not intended as validated numerical technology-readiness scores. Translational strengths and bottlenecks depend on the target mycotoxin, enzyme class, intended food or feed application, and available evidence.

**Table 8:** Representative computational approaches and their roles in Designer Enzyme development.

Engineering Task	Representative Approaches	Primary Contribution	Example Translational Question	Representative References
Candidate discovery	Genome mining, sequence comparison	Identification of potential detoxification enzymes and alternative catalytic scaffolds	Are additional enzyme scaffolds available for the target reaction?	[22]
Structural interpretation	Experimental structures, structure prediction, molecular modeling	Identification of catalytic residues, substrate-recognition regions, and structural constraints	Which residues control toxin recognition or catalysis?	[18,30]
Mutation prioritization	Structure-guided analysis, sequence-based prediction, machine-learning-assisted screening	Reduction in experimental search space and prioritization of promising variants	Which variants should be tested first?	[19,91]
Stability optimization	Structure-guided stability design, computational screening, MD-assisted analysis	Identification of substitutions or structural changes associated with improved stability	Can the enzyme tolerate processing-related thermal or pH stress?	[69,79]
Multi-objective engineering	Predictive models combined with experimental feedback	Coordinated optimization of activity, stability, selectivity, expression, or other application-relevant traits	Can one trait be improved without unacceptable loss of another?	[70]
Iterative refinement	DBTL workflows	Integration of prediction, experimentation, and learning across successive engineering cycles	What should be redesigned after each testing cycle?	[20]

The choice of computational approach should be determined by the dominant engineering bottleneck rather than by technological complexity alone.

**Table 9:** Application-specific regulatory considerations for mycotoxin-detoxifying enzymes.

Application	Representative Regulatory Context	Key Evidence Categories	Major Deployment Questions	Reference
Food processing	Food-enzyme/food-processing regulatory pathway	Production-organism safety, enzyme preparation, intended technological use, dietary exposure, reaction-product safety	Does the enzyme and detoxification process remain safe for the intended food use?	[115]
Feed application	Feed-additive regulatory pathway	Target-animal safety, efficacy, production consistency, degradation products, consumer and environmental safety	Does the enzyme safely and consistently reduce mycotoxin risk under intended feed conditions?	[115]
EU	EFSA-related evaluation under applicable food/feed legislation	Safety, efficacy, manufacturing, environmental considerations where applicable	Are application-specific regulatory requirements satisfied?	[115]
United States	FDA and application-specific pathways	Intended use, safety, manufacturing and efficacy evidence as applicable	What regulatory category applies to the intended product?	[121]
China	Relevant food/feed regulatory authorities	Registration, safety, efficacy, manufacturing quality	Are domestic registration and safety requirements satisfied?	[119]
Japan	Relevant food/feed safety authorities	Intended-use safety and application-specific assessment	Does the product meet local food/feed requirements?	[120]

Specific requirements depend on product classification, intended use, jurisdiction, and regulatory updates. Regulatory guidance should therefore be consulted for each proposed application rather than inferred from a general international comparison.

## Data Availability

No new data were created or analyzed in this study. Data sharing is not applicable to this article.

## References

[1] Eskola M., Kos G., Elliott C.T., Hajšlová J., Mayar S., Krska R. (2020). Worldwide contamination of food-crops with mycotoxins: Validity of the widely cited ‘FAO estimate’ of 25%. Crit. Rev. Food Sci. Nutr..

[2] Kovač Tomas M., Jurčević Šangut I. (2025). New Insights into Mycotoxin Contamination, Detection, and Mitigation in Food and Feed Systems. Toxins.

[3] Goda A.A., Shi J., Xu J., Liu X., Zhou Y., Xiao L., Abdel-Galil M., Salem S.H., Ayad E.G., Deabes M. (2025). Global health and economic impacts of mycotoxins: A comprehensive review. Environ. Sci. Eur..

[4] Khan R. (2024). Mycotoxins in food: Occurrence, health implications, and control strategies-A comprehensive review. Toxicon.

[5] Zingales V., Taroncher M., Martino P.A., Ruiz M.-J., Caloni F. (2022). Climate Change and Effects on Molds and Mycotoxins. Toxins.

[6] Li L., He Z., Shi Y., Sun H., Yuan B., Cai J., Chen J., Long M. (2023). Role of epigenetics in mycotoxin toxicity: A review. Environ. Toxicol. Pharmacol..

[7] Alassane-Kpembi I., Schatzmayr G., Taranu I., Marin D., Puel O., Oswald I.P. (2017). Mycotoxins co-contamination: Methodological aspects and biological relevance of combined toxicity studies. Crit. Rev. Food Sci. Nutr..

[8] Mwabulili F., Li P., Shi J., Zhang H., Xie Y., Ma W., Sun S., Yang Y., Li Q., Li X. (2024). Research diversity and advances in simultaneous removal of multi-mycotoxin. Toxicon.

[9] Nahle S., El Khoury A., Savvaidis I., Chokr A., Louka N., Atoui A. (2022). Detoxification approaches of mycotoxins: By microorganisms, biofilms and enzymes. Int. J. Food Contam..

[10] Lach M., Kotarska K. (2024). Negative Effects of Occurrence of Mycotoxins in Animal Feed and Biological Methods of Their Detoxification: A Review. Molecules.

[11] Ouyang B., Zhang W., Guang C., Xu W., Mu W. (2024). Opportunities and challenges in developing promising mycotoxin-degrading enzymes with high thermostability and strong pH activity/stability. Food Biosci..

[12] Liu L., Xie M., Wei D. (2022). Biological Detoxification of Mycotoxins: Current Status and Future Advances. Int. J. Mol. Sci..

[13] He J., Zhou T., Young J.C., Boland G.J., Scott P.M. (2010). Chemical and biological transformations for detoxification of trichothecene mycotoxins in human and animal food chains: A review. Trends Food Sci. Technol..

[14] Ismail A.M., Raza M.H., Zahra N., Ahmad R., Sajjad Y., Khan S.A. (2024). Aflatoxins in Wheat Grains: Detection and Detoxification through Chemical, Physical, and Biological Means. Life.

[15] Yao J., Ouyang B., Xu W., Xie Y., Mu W. (2026). An overview of the physical, chemical and biological strategies for the removal of emerging mycotoxins: Recent advances and future perspectives. Food Control.

[16] Adegoke T.V., Yang B., Xing F., Tian X., Wang G., Tai B., Si P., Hussain S., Jahan I. (2023). Microbial Enzymes Involved in the Biotransformation of Major Mycotoxins. J. Agric. Food Chem..

[17] Adegoke T.V., Lu S., Adegoke O.G., Wang Y., Wang Y. (2025). Optimizing single and cascade microbial enzyme systems through site-directed mutagenesis for enhancing mycotoxin detoxification. Biotechnol. Adv..

[18] Jumper J., Evans R., Pritzel A., Green T., Figurnov M., Ronneberger O., Tunyasuvunakool K., Bates R., Žídek A., Potapenko A. (2021). Highly accurate protein structure prediction with AlphaFold. Nature.

[19] Yang K.K., Wu Z., Arnold F.H. (2019). Machine-learning-guided directed evolution for protein engineering. Nat. Methods.

[20] HamediRad M., Chao R., Weisberg S., Lian J., Sinha S., Zhao H. (2019). Towards a fully automated algorithm driven platform for biosystems design. Nat. Commun..

[21] Meng F., Ellis T. (2020). The second decade of synthetic biology: 2010–2020. Nat. Commun..

[22] Shi J., Mwabulili F., Xie Y., Yang Y., Sun S., Li Q., Ma W., Jia H. (2024). Characterization, Structural Analysis, and Thermal Stability Mutation of a New Zearalenone-Degrading Enzyme Mined from *Bacillus subtilis*. J. Agric. Food Chem..

[23] Bampidis V., Azimonti G., Bastos M.D.L., Christensen H., Dusemund B., Kos Durjava M., Kouba M., López-Alonso M., López Puente S., EFSA Panel on Additives and Products or Substances Used in Animal Feed (FEEDAP) (2020). Safety and efficacy of fumonisin esterase from *Komagataella phaffii* DSM 32159 as a feed additive for all animal species. EFSA J..

[24] EFSA Panel on Additives and Products or Substances Used in Animal Feed (FEEDAP) (2014). Scientific Opinion on the safety and efficacy of fumonisin esterase (FUMzyme^®^) as a technological feed additive for pigs. EFSA J..

[25] Sun H., He Z., Xiong D., Long M. (2023). Mechanisms by which microbial enzymes degrade four mycotoxins and application in animal production: A review. Anim. Nutr..

[26] Takahashi-Ando N., Kimura M., Kakeya H., Osada H., Yamaguchi I. (2002). A novel lactonohydrolase responsible for the detoxification of zearalenone: Enzyme purification and gene cloning. Biochem. J..

[27] Fruhauf S., Novak B., Nagl V., Hackl M., Hartinger D., Rainer V., Labudová S., Adam G., Aleschko M., Moll W.-D. (2019). Biotransformation of the Mycotoxin Zearalenone to its Metabolites Hydrolyzed Zearalenone (HZEN) and Decarboxylated Hydrolyzed Zearalenone (DHZEN) Diminishes its Estrogenicity In Vitro and In Vivo. Toxins.

[28] Yao Y., Long M. (2020). The biological detoxification of deoxynivalenol: A review. Food Chem. Toxicol..

[29] Zhang X., Ouyang B., Xu W., Sun H., Mu W. (2026). Recent advances in microbial laccases for aflatoxins control: Identification, modification, and application. Int. J. Biol. Macromol..

[30] Dai L., Niu D., Huang J.-W., Li X., Shen P., Li H., Xie Z., Min J., Hu Y., Yang Y. (2023). Cryo-EM structure and rational engineering of a superefficient ochratoxin A-detoxifying amidohydrolase. J. Hazard. Mater..

[31] Sánchez-Arroyo A., Plaza-Vinuesa L., Rivas B.D.L., Mancheño J.M., Muñoz R. (2023). The salicylate 1,2-dioxygenase from *Pseudaminobacter salicylatoxidans* DSM 6986T is a bifunctional enzyme that inactivates the mycotoxin ochratoxin A by a novel amidohydrolase activity. Int. J. Biol. Macromol..

[32] Heinl S., Hartinger D., Thamhesl M., Vekiru E., Krska R., Schatzmayr G., Moll W.-D., Grabherr R. (2010). Degradation of fumonisin B1 by the consecutive action of two bacterial enzymes. J. Biotechnol..

[33] Alberts J., Schatzmayr G., Moll W.-D., Davids I., Rheeder J., Burger H.-M., Shephard G., Gelderblom W. (2019). Detoxification of the Fumonisin Mycotoxins in Maize: An Enzymatic Approach. Toxins.

[34] Liu M., Zhang X., Luan H., Zhang Y., Xu W., Feng W., Song P. (2024). Bioenzymatic detoxification of mycotoxins. Front. Microbiol..

[35] Wang H., Sun S., Ge W., Zhao L., Kong L. (2020). Horizontal gene transfer of Fhb7 from fungus underlies Fusarium head blight resistance in wheat. Science.

[36] Incze D.J., Molnár Z., Nagy G.N., Leveles I., Vértessy B.G., Poppe L., Bata Z. (2025). Understanding the molecular mechanism of fumonisin esterases by kinetic and structural studies. Food Chem..

[37] Loi M., Fanelli F., Liuzzi V.C., Logrieco A.F., Mulè G. (2017). Mycotoxin Biotransformation by Native and Commercial Enzymes: Present and Future Perspectives. Toxins.

[38] Karlovsky P., Suman M., Berthiller F., De Meester J., Eisenbrand G., Perrin I., Oswald I.P., Speijers G., Chiodini A., Recker T. (2016). Impact of food processing and detoxification treatments on mycotoxin contamination. Mycotoxin Res..

[39] Bullerman L.B., Bianchini A. (2007). Stability of mycotoxins during food processing. Int. J. Food Microbiol..

[40] Yang X., Yao M., Liao W., Li X. (2025). Adsorption–Degradation Integrated Approaches to Mycotoxin Removal from Food Matrices: A Comprehensive Review. Toxins.

[41] Aasa A.O., Govender S.E., Malgas S., Thantsha M.S. (2026). Microbial and enzymatic biodegradation of aflatoxins and ochratoxins: Mechanisms, applications, and emerging innovations. Arch. Microbiol..

[42] Schaarschmidt S., Fauhl-Hassek C. (2018). The Fate of Mycotoxins During the Processing of Wheat for Human Consumption. Compr. Rev. Food Sci. Food Saf..

[43] Shi Y., Ouyang B., Zhang Y., Zhang W., Xu W., Mu W. (2024). Recent developments of mycotoxin-degrading enzymes: Identification, preparation and application. Crit. Rev. Food Sci. Nutr..

[44] Zhang H., Zhang S., Ma W., Yang Y., Li X., Xie Y. (2025). Amphiphilicity and thermostability modification of dipeptidyl peptidase III from *aspergillus terreus* HNGD-TM15 capable of simultaneously degrading aflatoxin B1 and zearalenone. Food Chem..

[45] Niu J., Ma B., Shen J., Zhu H., Lu Y., Lu Z., Lu F., Zhu P. (2025). Enzymatic degradation of mycotoxin patulin by a short-chain dehydrogenase/reductase from *Bacillus subtilis* and its application in apple juice. Food Microbiol..

[46] Yang C., Hu C., Huang L., Peng B. (2025). Mechanism of patulin biodegradation by a reductase from *Saccharomyces cerevisiae* and its potential application to apple juice. Food Res. Int..

[47] Xing M., Chen Y., Li B., Tian S. (2021). Characterization of a short-chain dehydrogenase/reductase and its function in patulin biodegradation in apple juice. Food Chem..

[48] Inborr J., Bedford M.R. (1994). Stability of feed enzymes to steam pelleting during feed processing. Anim. Feed Sci. Technol..

[49] Morgavi D.P., Beauchemin K.A., Nsereko V.L., Rode L.M., McAllister T.A., Iwaasa A.D., Wang Y., Yang W.Z. (2001). Resistance of feed enzymes to proteolytic inactivation by rumen microorganisms and gastrointestinal proteases. J. Anim. Sci..

[50] Nobre C., González A., Losoya C., Teixeira J.A., Belmares R., Abrunhosa L. (2022). Detoxification of ochratoxin A and zearalenone by Pleurotus ostreatus during in vitro gastrointestinal digestion. Food Chem..

[51] Ning Y.-Q., Peng Z., Zhang Y., Refaie A., Ge J.-H., Guo L.-J., Yang W.-J., Sun L.-H. (2025). A novel mycotoxin-degrading enzyme complex can biodegrade AFB1, DON, and ZEN co-contamination in both in vitro and in vivo experiments. Toxicon.

[52] Alberts J.F., Davids I., Moll W.-D., Schatzmayr G., Burger H.-M., Shephard G.S., Gelderblom W.C.A. (2021). Enzymatic detoxification of the fumonisin mycotoxins during dry milling of maize. Food Control.

[53] Grenier B., Schwartz-Zimmermann H.E., Gruber-Dorninger C., Dohnal I., Aleschko M., Schatzmayr G., Moll W.D., Applegate T.J. (2017). Enzymatic hydrolysis of fumonisins in the gastrointestinal tract of broiler chickens. Poult. Sci..

[54] Gruber-Dorninger C., Aleschko M., Höbartner-Gußl A., Fruhauf S., Thamhesl M., Doupovec B., Schatzmayr D., Moll W.-D., Pender C. (2025). Review article: Recent advances in enzyme technologies for mitigating mycotoxin contamination in poultry feed. J. Appl. Poult. Res..

[55] Robic S., Linscott K.B., Aseem M., Humphreys E.A., McCartha S.R. (2011). Bile Acids as Modulators of Enzyme Activity and Stability. Protein J..

[56] Kurniawan D.C., Rohman M.S., Witasari L.D. (2024). Heterologous expression, characterization, and application of recombinant thermostable α-amylase from *Geobacillus* sp. DS3 for porous starch production. Biochem. Biophys. Rep..

[57] Mwabulili F., Xie Y., Li Q., Sun S., Yang Y., Ma W. (2023). Research progress of ochratoxin a bio-detoxification. Toxicon.

[58] Binder S.B., Schwartz-Zimmermann H.E., Varga E., Bichl G., Michlmayr H., Adam G., Berthiller F. (2017). Metabolism of Zearalenone and Its Major Modified Forms in Pigs. Toxins.

[59] Michlmayr H., Varga E., Lupi F., Malachová A., Hametner C., Berthiller F., Adam G. (2017). Synthesis of Mono- and Di-Glucosides of Zearalenone and α-/β-Zearalenol by Recombinant Barley Glucosyltransferase HvUGT14077. Toxins.

[60] Zhang Z., Cai Y., Fan K., Huang Q., Zhao X., Cao H., Zhao Z., Tangni E.K., Han Z. (2022). Development of a reliable UHPLC-MS/MS method for simultaneous determination of zearalenone and zearalenone-14-glucoside in various feed products. Front. Chem..

[61] Hou B., Xu Y., Wang D., Dong C., Yan F., Cheng X., Sun S., Bo C., Liu Z., Qin Y. (2024). A novel strategy for detoxification of deoxynivalenol via modification of both toxic groups. Food Chem..

[62] Niu J., Yan R., Zhou H., Ma B., Lu Z., Meng F., Lu F., Zhu P. (2024). Self-cascade deoxynivalenol detoxification by an artificial enzyme with bifunctions of dehydrogenase and aldo/keto reductase from genome mining. Int. J. Biol. Macromol..

[63] Zhang H., Du X., Wu X., Zhang H., Guo Z., Nie Z., Li X., Delidovich I., Lee S.J., Xia Y. (2026). Construction and evaluation of a bifunctional fusion enzyme for cascade degradation of deoxynivalenol. Int. J. Biol. Macromol..

[64] Bampidis V., Azimonti G., Bastos M.D.L., Christensen H., Dusemund B., Kos Durjava M., Kouba M., López-Alonso M., López Puente S., EFSA Panel on Additives and Products or Substances Used in Animal Feed (FEEDAP) (2018). Safety and efficacy of fumonisin esterase from *Komagataella phaffii* DSM 32159 as a technological feed additive for pigs and poultry. EFSA J..

[65] Ndochinwa O.G., Wang Q.-Y., Amadi O.C., Nwagu T.N., Nnamchi C.I., Okeke E.S., Moneke A.N. (2024). Current status and emerging frontiers in enzyme engineering: An industrial perspective. Heliyon.

[66] Ndochinwa G.O., Wang Q.-Y., Okoro N.O., Amadi O.C., Nwagu T.N., Nnamchi C.I., Moneke A.N., Odiba A.S. (2024). New advances in protein engineering for industrial applications: Key takeaways. Open Life Sci..

[67] Bergeson A.R., Alper H.S. (2024). Advancing sustainable biotechnology through protein engineering. Trends Biochem. Sci..

[68] Albayati S.H., Nezhad N.G., Taki A.G., Rahman R.N.Z.R.A. (2024). Efficient and easible biocatalysts: Strategies for enzyme improvement. A review. Int. J. Biol. Macromol..

[69] Jiang X., Tehreem S., Rahim K., Wang M., Wu P., Zhang G. (2024). Enhancing the thermal stability and activity of zearalenone lactone hydrolase to promote zearalenone degradation via semi-rational design. Enzym. Microb. Technol..

[70] Teufl M., Zajc C.U., Traxlmayr M.W. (2022). Engineering Strategies to Overcome the Stability–Function Trade-Off in Proteins. ACS Synth. Biol..

[71] Nanda V., Belure S.V., Shir O.M. (2017). Searching for the Pareto frontier in multi-objective protein design. Biophys. Rev..

[72] Sayed H., Tang Y., Guo Y., Zhao L. (2026). Zearalenone and its major modified forms: A comprehensive review of biosynthesis, toxicity, metabolism, and biodegradation strategies for food safety applications. Food Chem. Toxicol..

[73] Arimboor R. (2024). Metabolites and degradation pathways of microbial detoxification of aflatoxins: A review. Mycotoxin Res..

[74] Luo H., Wang G., Chen N., Fang Z., Xiao Y., Zhang M., Gerelt K., Qian Y., Lai R., Zhou Y. (2022). A Superefficient Ochratoxin A Hydrolase with Promising Potential for Industrial Applications. Appl. Environ. Microbiol..

[75] Ding L., Han M., Wang X., Guo Y. (2023). Ochratoxin A: Overview of Prevention, Removal, and Detoxification Methods. Toxins.

[76] Li Z., Wang Y., Liu Z., Jin S., Pan K., Liu H., Liu T., Li X., Zhang C., Luo X. (2021). Biological detoxification of fumonisin by a novel carboxylesterase from *Sphingomonadales bacterium* and its biochemical characterization. Int. J. Biol. Macromol..

[77] Schertz H., Kluess J., Frahm J., Schatzmayr D., Dohnal I., Bichl G., Schwartz-Zimmermann H., Breves G., Dänicke S. (2018). Oral and Intravenous Fumonisin Exposure in Pigs—A Single-Dose Treatment Experiment Evaluating Toxicokinetics and Detoxification. Toxins.

[78] Xu Z., Liu W., Chen C.-C., Li Q., Huang J.-W., Ko T.-P., Liu G., Liu W., Peng W., Cheng Y.-S. (2016). Enhanced α-Zearalenol Hydrolyzing Activity of a Mycoestrogen-Detoxifying Lactonase by Structure-Based Engineering. ACS Catal..

[79] Guan A., Zhang M., Xu F. (2023). Structure-guided engineering for improving the thermal stability of zearalenone hydrolase. Chin. J. Biotechnol..

[80] Hou B., Wang D., Yan F., Cheng X., Xu Y., Xi X., Ge W., Sun S., Su P., Zhao L. (2024). Fhb7-GST catalyzed glutathionylation effectively detoxifies the trichothecene family. Food Chem..

[81] Guo Y., Qin X., Tang Y., Ma Q., Zhang J., Zhao L. (2020). CotA laccase, a novel aflatoxin oxidase from *Bacillus licheniformis*, transforms aflatoxin B1 to aflatoxin Q1 and epi-aflatoxin Q1. Food Chem..

[82] Liu Y., Liu L., Huang Z., Guo Y., Tang Y., Wang Y., Ma Q., Zhao L. (2024). Combined Strategies for Improving Aflatoxin B1 Degradation Ability and Yield of a *Bacillus licheniformis* CotA-Laccase. Int. J. Mol. Sci..

[83] Wang X., Bai Y., Huang H., Tu T., Wang Y., Wang Y., Luo H., Yao B., Su X. (2019). Degradation of Aflatoxin B1 and Zearalenone by Bacterial and Fungal Laccases in Presence of Structurally Defined Chemicals and Complex Natural Mediators. Toxins.

[84] Gao B., An W., Wu J., Wang X., Han B., Tao H., Liu J., Wang Z., Wang J. (2024). Simultaneous Degradation of AFB1 and ZEN by CotA Laccase from *Bacillus subtilis* ZJ-2019-1 in the Mediator-Assisted or Immobilization System. Toxins.

[85] Zeinvand-Lorestani H., Sabzevari O., Setayesh N., Amini M., Nili-Ahmadabadi A., Faramarzi M.A. (2015). Comparative study of in vitro prooxidative properties and genotoxicity induced by aflatoxin B1 and its laccase-mediated detoxification products. Chemosphere.

[86] Liu Y., Mao H., Woldemariam Yohannes K., Wan Z., Cao Y., Tron T., Lin J., Jiang Y., Li H., Wang J. (2021). Degradation of aflatoxin B1 by a recombinant laccase from *Trametes* sp. C30 expressed in *Saccharomyces cerevisiae*: A mechanism assessment study in vitro and in vivo. Food Res. Int..

[87] Liu B., Xiong M., Sun W., Wu H., Li J., Wang Y., Wang Q., He J., Chen X. (2026). Biochemical characterization of a novel ochratoxin A-detoxifying amidohydrolase from *Terribacillus saccharophilus* JX-8 and its application in grape juice and wheat flour. Food Res. Int..

[88] Masching S., Naehrer K., Schwartz-Zimmermann H.-E., Sărăndan M., Schaumberger S., Dohnal I., Nagl V., Schatzmayr D. (2016). Gastrointestinal Degradation of Fumonisin B1 by Carboxylesterase FumD Prevents Fumonisin Induced Alteration of Sphingolipid Metabolism in Turkey and Swine. Toxins.

[89] Hu Y., Xu Y., He B., Niu D., Yang X., Huang J., Xie Z., Shen P., Li X., Bai M. (2026). Structure-guided protein engineering and immobilization of an amidohydrolase for efficient ochratoxin A detoxification. Int. J. Biol. Macromol..

[90] Watson J.L., Juergens D., Bennett N.R., Trippe B.L., Yim J., Eisenach H.E., Ahern W., Borst A.J., Ragotte R.J., Milles L.F. (2023). De novo design of protein structure and function with RFdiffusion. Nature.

[91] Dauparas J., Anishchenko I., Bennett N., Bai H., Ragotte R.J., Milles L.F., Wicky B.I.M., Courbet A., de Haas R.J., Bethel N. (2022). Robust deep learning-based protein sequence design using ProteinMPNN. Science.

[92] Terwilliger T.C., Liebschner D., Croll T.I., Williams C.J., McCoy A.J., Poon B.K., Afonine P.V., Oeffner R.D., Richardson J.S., Read R.J. (2024). AlphaFold predictions are valuable hypotheses and accelerate but do not replace experimental structure determination. Nat. Methods.

[93] Xing X., Chen X., You X., Huang J., Xue D. (2024). Zearalenone degrading enzyme evolution to increase the hydrolysis efficiency under acidic conditions by the rational design. Food Chem..

[94] Madani A., Krause B., Greene E.R., Subramanian S., Mohr B.P., Holton J.M., Olmos J.L., Xiong C., Sun Z.Z., Socher R. (2023). Large language models generate functional protein sequences across diverse families. Nat. Biotechnol..

[95] Unsal S., Atas H., Albayrak M., Turhan K., Acar A.C., Doğan T. (2022). Learning functional properties of proteins with language models. Nat. Mach. Intell..

[96] Goldenzweig A., Goldsmith M., Hill S.E., Gertman O., Laurino P., Ashani Y., Dym O., Unger T., Albeck S., Prilusky J. (2016). Automated Structure- and Sequence-Based Design of Proteins for High Bacterial Expression and Stability. Mol. Cell.

[97] Wijma H.J., Floor R.J., Jekel P.A., Baker D., Marrink S.J., Janssen D.B. (2014). Computationally designed libraries for rapid enzyme stabilization. Protein Eng. Des. Sel..

[98] Peleg Y., Vincentelli R., Collins B.M., Chen K.-E., Livingstone E.K., Weeratunga S., Leneva N., Guo Q., Remans K., Perez K. (2021). Community-Wide Experimental Evaluation of the PROSS Stability-Design Method. J. Mol. Biol..

[99] Duman-Özdamar Z.E., Binay B. (2021). Production of Industrial Enzymes via Pichia pastoris as a Cell Factory in Bioreactor: Current Status and Future Aspects. Protein J..

[100] Waegeman H., Soetaert W. (2011). Increasing recombinant protein production in *Escherichia coli* through metabolic and genetic engineering. J. Ind. Microbiol. Biotechnol..

[101] Rosano G.L., Ceccarelli E.A. (2014). Recombinant protein expression in *Escherichia coli*: Advances and challenges. Front. Microbiol..

[102] Mital S., Christie G., Dikicioglu D. (2021). Recombinant expression of insoluble enzymes in *Escherichia coli*: A systematic review of experimental design and its manufacturing implications. Microb. Cell Fact..

[103] Bhatwa A., Wang W., Hassan Y.I., Abraham N., Li X.-Z., Zhou T. (2021). Challenges Associated With the Formation of Recombinant Protein Inclusion Bodies in *Escherichia coli* and Strategies to Address Them for Industrial Applications. Front. Bioeng. Biotechnol..

[104] Yoon S.H., Kim S.K., Kim J.F. (2010). Secretory Production of Recombinant Proteins in *Escherichia coli*. Recent Pat. Biotechnol..

[105] Lokireddy S.R., Kunchala S.R., Vadde R. (2025). Advancements in *Escherichia coli* secretion systems for enhanced recombinant protein production. World J. Microbiol. Biotechnol..

[106] Pokoo-Aikins A., McDonough C.M., Mitchell T.R., Hawkins J.A., Adams L.F., Read Q.D., Li X., Shanmugasundaram R., Rodewald E., Acharya P. (2024). Mycotoxin contamination and the nutritional content of corn targeted for animal feed. Poult. Sci..

[107] Gomes A.L., Petrus R.R., de Sousa R.L.M., Fernandes A.M. (2024). Aflatoxins and fumonisins in conventional and organic corn: A comprehensive review. Food Addit. Contam. Part A.

[108] Berthiller F., Crews C., Dall’Asta C., Saeger S.D., Haesaert G., Karlovsky P., Oswald I.P., Seefelder W., Speijers G., Stroka J. (2013). Masked mycotoxins: A review. Mol. Nutr. Food Res..

[109] Wu W., Lu S., Jiang S., Chen J., Zheng Z., Jiang S., Yang P. (2024). Immobilization of recombinant *Trametes versicolor* aflatoxin B1-degrading enzyme (TV-AFB1D) with montmorillonite for absorption and in situ degradation of aflatoxin B1. Mycotoxin Res..

[110] Zhou F., Liu Q., Chen K., Du M., Zalán Z., Awad S., Kan J. (2026). Identification of a novel patulin degrading enzyme from *Wickerhamomyces anomalus* XL1 and its application in apple juice. Food Chem..

[111] Zhou F., Lv Y., Liu Q., Chen K., Du M., Zalán Z., Awad S., Kan J. (2026). A new Patulin-degrading enzyme from *Wickerhamomyces anomalus* XL1: Identification, mechanism, and application. Int. J. Food Microbiol..

[112] Charmley L.L., Trenholm H.L., Prelusky D.B., Rosenberg A. (1995). Economic losses and decontamination. Nat. Toxins.

[113] Osweiler G.D. (2000). Mycotoxins: Contemporary Issues of Food Animal Health and Productivity. Veter Clin. N. Am. Food Anim. Pract..

[114] Nachtergaele P., Kocak O., Roman Escobar Y., Motte J., Gabriels D., Mottet L., Dewulf J. (2024). Does enzymatic catalysis lead to more sustainable chemicals production? A life cycle sustainability assessment of isopropyl palmitate. Green Chem..

[115] Regulation (EC) No 1831/2003 of the European Parliament and of the Council of 22 September 2003 on Additives for Use in Animal Nutrition. 1831. https://eur-lex.europa.eu/eli/reg/2003/1831/oj.

[116] Aids P., Lambré C., Barat Baviera J.M., Bolognesi C., Cocconcelli P.S., Crebelli R., Gott D.M., Grob K., Lampi E., EFSA Panel on Food Contact Materials (2021). Scientific Guidance for the submission of dossiers on Food Enzymes. EFSA J..

[117] Commission Regulation (EC) No 386/2009 of 12 May 2009 Amending Regulation (EC) No 1831/2003 as Regards the Establishment of a New Functional Group of Feed Additives. http://data.europa.eu/eli/reg/2009/386/oj.

[118] (2010). Guidance for Industry: Recommendations for Submission of Chemical and Technological Data for Food Additive Petitions and GRAS Notices for Enzyme Preparations. https://www.fda.gov/regulatory-information/search-fda-guidance-documents/guidance-industry-recommendations-submission-chemical-and-technological-data-food-additive-petitions.

[119] (2017). Regulation on the Administration of Feed and Feed Additives. https://www.samr.gov.cn/zw/zfxxgk/fdzdgknr/bgt/art/2023/art_8d361ca1bdee414f99f9fc2bd0903c82.html.

[120] Ministry of Health, Labour and Welfare, Japan Food Additives. https://www.mhlw.go.jp/stf/seisakunitsuite/bunya/kenkou_iryou/shokuhin/syokuten/index_00012.html.

[121] U.S. Food and Drug Administration (2018). Enzyme Preparations Used in Food (Partial List). https://www.fda.gov/food/generally-recognized-safe-gras/enzyme-preparations-used-food-partial-list.

[122] Zhang Q., Chen W., Qin M., Wang Y., Pu Z., Ding K., Liu Y., Zhang Q., Li D., Li X. (2025). Integrating protein language models and automatic biofoundry for enhanced protein evolution. Nat. Commun..

[123] Singh N., Lane S., Yu T., Lu J., Ramos A., Cui H., Zhao H. (2025). A generalized platform for artificial intelligence-powered autonomous enzyme engineering. Nat. Commun..

[124] Purlis E. (2024). Digital Twin Methodology in Food Processing: Basic Concepts and Applications. Curr. Nutr. Rep..

